# Nanoscopic Insights of Amphiphilic Peptide against the Oligomer Assembly Process to Treat Huntington's Disease

**DOI:** 10.1002/advs.201901165

**Published:** 2019-12-09

**Authors:** Ruei‐Yu He, Xiang‐Me Lai, Chia‐Sui Sun, Te‐Shien Kung, Jhu‐Ying Hong, Yu‐Song Jheng, Wei‐Neng Liao, Jen‐Kun Chen, Yung‐Feng Liao, Pang‐Hsien Tu, Joseph Jen‐Tse Huang

**Affiliations:** ^1^ Institute of Chemistry Academia Sinica Taipei 11529 Taiwan; ^2^ Institute of Biomedical Sciences Academia Sinica Taipei 11529 Taiwan; ^3^ Division of Urology Department of Surgery Tri‐Service General Hospital National Defense Medical Center Taipei 11490 Taiwan; ^4^ Department of Chemical Engineering National Taiwan University of Science and Technology Taipei 10607 Taiwan; ^5^ Institute of Biomedical Engineering and Nanomedicine National Health Research Institutes Miaoli 35053 Taiwan; ^6^ Institute of Cellular and Organismic Biology Academia Sinica Taipei 11529 Taiwan

**Keywords:** amphiphilic peptides, fluorescence lifetime imaging, Huntington's disease, intranasal administration, oligomer assembly

## Abstract

Finding an effective therapeutic regimen is an urgent demand for various neurodegenerative disorders including Huntington's disease (HD). For the difficulties in observing the dynamic aggregation and oligomerization process of mutant Huntingtin (mHtt) in vivo, the evaluation of potential drugs at the molecular protein level is usually restricted. By combing lifetime‐based fluorescence microscopies and biophysical tools, it is showcased that a designed amphiphilic peptide, which targets the mHtt at an early stage, can perturb the oligomer assembly process nanoscopically, suppress the amyloid property of mHtt, conformationally transform the oligomers and/or aggregates of mHtt, and ameliorate mHtt‐induced neurological damage and aggregation in cell and HD mouse models. It is also found that this amphiphilic peptide is able to transport to the brain and rescue the memory deficit through intranasal administration, indicating its targeting specificity in vivo. In summary, a biophotonic platform is provided to investigate the oligomerization/aggregation process in detail that offers insight into the design and effect of a targeted therapeutic agent for Huntington's disease.

## Graphical abstract

An amphiphilic peptide (8R10Q) that assembles into nanovesicles and targets mutant Huntingtin (mHtt) protein is designed. By applying various advanced nanoscopic tools, it is shown that 8R10Q perturbs the mHtt oligomer assembly process, conformationally transforms mHtt inclusions into puncta, and reduces mHtt toxicity. Moreover, 8R10Q can be transported into the brain to ameliorate mHtt‐induced neurological damage and rescue memory deficit through intranasal administration.

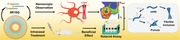

## 1 Introduction

Neurodegenerative diseases such as Huntington's disease (HD), Alzheimer's disease (AD), and Parkinson's disease (PD) are categorized as protein‐misfolding diseases for their shared feature of inclusion bodies formed by aggregation of misfolded proteins.^1^ Within these cases, HD is an autosomal dominantly inherited neurodegenerative disease caused by an expansion of the CAG trinucleotide repeats in the gene *Huntingtin* (*HTT*), which encodes a mutant Huntingtin (mHtt) protein containing a prolonged polyglutamine (polyQ) stretch.^2^ In HD, the prolonged polyQ confers to the mHtt protein an aberrant propensity for self‐aggregation to form neurotoxic species, be it an oligomer^3^ or fibrillary aggregate,^4^ which are linked with HD pathogenesis. No efficacious disease‐modifying treatment is currently available.

Conceptually, reducing the toxic species or toxic species formed by the misfolded protein or its derivative may be a practical and safe therapeutic strategy.^5^ A series of clinical trials based on this approach^6^ or other strategies such as antioxidation,^7^ anti‐inflammation,^8^ or neuroprotection^9^ fail to yield clinical benefit. Recently, misfolded protein with neuropathology has been shown to precede clinical symptoms even by decades.^10^ These trials based on clinical symptoms may have missed the best therapeutic window. Therefore, an emerging trend to tackle the neurodegenerative diseases is shifting from treating clinical disease to prevention in the preclinical stages.^11^

Previous studies have demonstrated that amyloid clusters formed under different conditions resulted in morphologically different and structurally distinct aggregate species, leading to distinct pathological phenotypes.^12^ Therefore, a sensitive method to monitor the dynamic aggregation/oligomerization process and evaluate the efficacy of the designed intervention strategy against HD is crucial. In this study, we combined advanced biophotonic approaches including frequency‐domain fluorescence lifetime imaging microscopy (FLIM) and quantitative fluorescence resonance energy transfer (FRET) efficiency by the phasor trajectory approach^13^ to provide conformational and morphological information of mHtt aggregates and/or intermediates. Fluorescence lifetime change induced by quenching or environmental change of the fluorophores was applied to reflect the structural change of amyloid clusters.^14^ This platform facilitated the selection of therapeutic agents by monitoring the efficacy of the treatment and provided detailed insight into the mechanisms by which the designed peptide modulated the mHtt fibrillization process.

To design a preventive strategy and evaluate its influences on mHtt aggregation and oligomerization process, an amphiphilic peptide (8R10Q) containing of positively charged arginine residues and a stretch of decaglutamines was chemically synthesized (sequence in Table S1 in the Supporting Information) and its therapeutic value was investigated in in vitro, cellular, and transgenic mouse models. This peptide showed great ability to form complex with the pathological mHtt protein, inhibit oligomerization and/or fibrillization process of the latter, and reduce the available endogenous proteins being sequestered during the fibrillization process. It also ameliorated mHtt‐induced neurotoxicity in vivo as monitored with various biophotonic tools, biochemical analysis, and biological assays.

## 2 Results

### 2.1 The Impact of the Amphiphilic Peptides toward the Oligomerization Process of polyQ‐Expanded mHtt in Cell

**Scheme 1:**
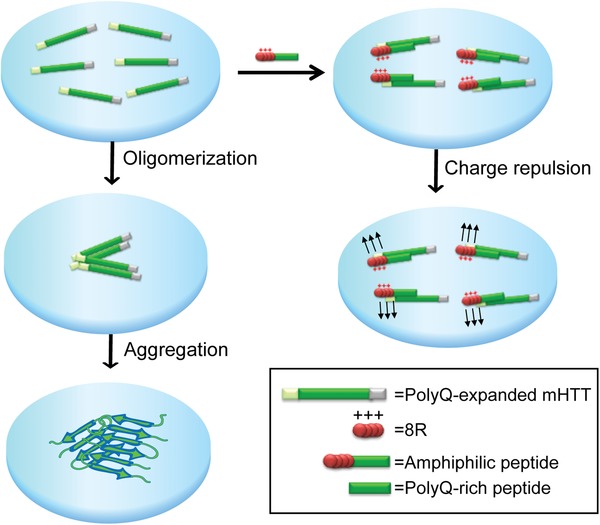
Schematic illustration of the modular design of the amphiphilic peptide against polyQ‐expanded mutant Huntingtin (mHtt) oligomerization and aggregation process in HD.

The design of the amphiphilic peptides was based on the principle of modular assembly of a bipartite structure harboring a self‐aggregating polyQ sequence of the mHtt attached to a positively charged polyarginines. The rationale behind was that the polyQ sequence would provide a specific and high affinity to the polyQ stretch in mHtt protein through its self‐aggregating property.^15^ The polyarginines were chosen for the following reasons. First of all, polyarginines stretch enabled the amphiphilic peptides to penetrate cell membrane.^16^ Second, polyarginines can increase solubility and prevent the amphiphilic peptides from aggregation. Lastly, after the peptide interacted with mHtt, it might also prevent the hybrids of the mHtt/amphiphilic peptide from self‐aggregation through charge repulsion (**Scheme** 1).

**Figure 1:**
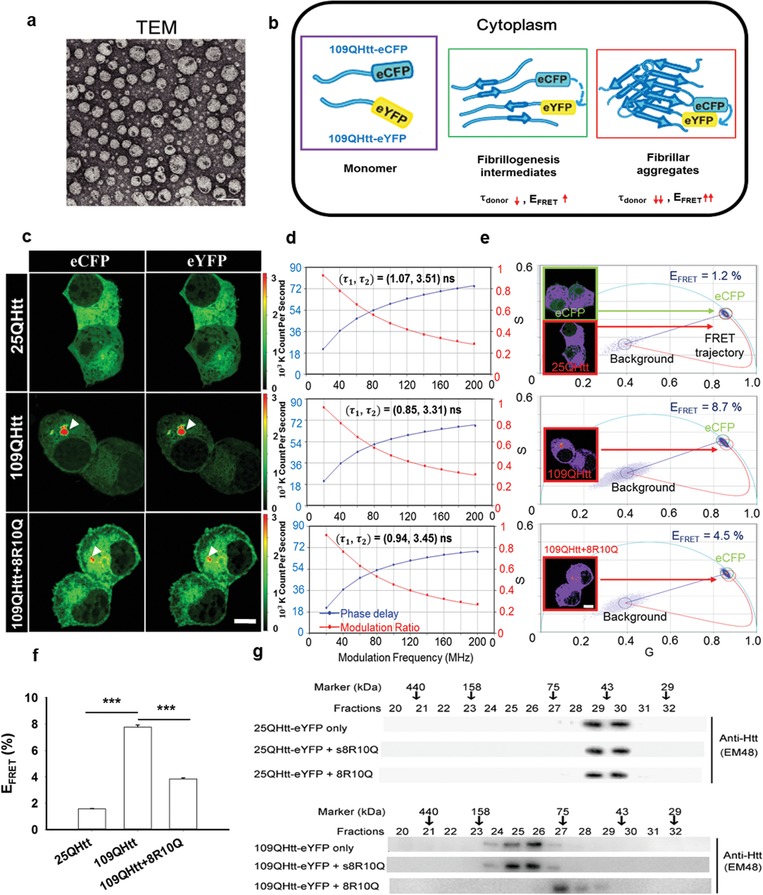
8R10Q decreased the compactness of mHtt oligomers and shifted the oligomerization process to form low‐ordered oligomers in Neuro2a cell. a) TEM imaging of the spherical vesicles from 0.5 × 10^−6^ m 8R10Q peptide. Scale bar: 100 nm. b) Schematics of the fibrillogenesis process resulted in decreased average fluorescence lifetime (τ_donor_) and increased FRET efficiency (*E*_FRET_) of the donor (eCFP). c) Neuro2a cells co‐expressing 25QHtt‐eCFP and 25Q‐eYFP (top), 109QHtt‐eCFP and 109QHtt‐eYFP (middle), and 109QHtt‐eCFP and 109QHtt‐eYFP in the presence of 8R10Q (bottom). d) Donor multifrequency FLIM data of the soluble fraction in panel (c) was fitted with the double‐exponential decay model. Additional information was summarized in Table S2. e) The phasor plot of the soluble fraction in panel (c) and the corresponding FRET efficiency (0–100%) from the curved trajectory. Pixels highlighted in purple correspond to the phasors within green or red circles. f) Quantitative FRET efficiency (*E*_FRET_) of the soluble fraction in the 25QHtt, 109QHtt, and 109QHtt treated with 8R10Q group by applying the FRET trajectory approach. All data were shown as mean ± standard error of the mean (SEM), *n* = 12. Statistics were done with one‐way ANOVA followed by Duncan's Multiple Range test. ***p < 0.001. g) Cell lysate from 25QHtt‐eYFP (top) or 109QHtt‐eYFP transfectants (bottom) treated with or without peptides was loaded into size exclusion column, and each fraction was analyzed by western blot using anti‐Huntingtin antibody (EM48). Scale bar: 10 µm.

The amphiphilic peptide, 8R10Q, was synthesized and characterized (sequence in Table S1 in the Supporting Information). Note that the tryptophan in these peptides was designed to facilitate the concentration quantification through its intrinsic absorbance and/or fluorescence. 8R10Q peptide was highly soluble in water or culture medium as anticipated (Table S1, Supporting Information) and self‐assembled into vesicles (**Figure** 1a) with a vesicle size of around 70 ± 15 nm (Figure S1, Supporting Information) under transmission electron microscopy (TEM) based on the principle of modular assembly of bipartite structure.[qv: 15b] The mean hydrodynamic diameter (average vesicle size) of 8R10Q was 96 ± 12 nm as characterized by dynamic light scattering (DLS) with a low polydispersity index (>0.3), indicating the formation of monodispersed nanovesicles (Figure S1, Supporting Information).

To test the potential of the amphiphilic peptide in tackling HD, we first tested its impact on the oligomerization process of mHtt in Neuro2a cell. Plasmids encoding N‐terminus Huntingtin exon 1 fragments harboring either nonpathological (25Q) or pathological (109Q) polyglutamine repeats fused with enhanced yellow fluorescent protein (eYFP) or enhanced cyan fluorescent protein (eCFP) were co‐transfected into cells (details in “Experimental Section”). 109Q‐eYFP and 109Q‐eCFP represent the mHtt protein as the prolonged polyQ stretch is prone to misfold and trigger the oligomerization and aggregation process. Taking advantage of FLIM in measuring the change of fluorescence lifetime of the donor involved in the energy transfer process, we tested if 8R10Q peptide altered the intramolecular/intermolecular interactions of mHtt oligomers and aggregates. Upon fibrillogenesis, mHtt‐eCFP (donor) and mHtt‐eYFP (acceptor) molecules interact with each other to form fibrillogenesis intermediates and subsequently assembled into fibrillar aggregate, which leads to increased FRET efficiency (*E*_FRET_) and decreased fluorescence lifetime of the donor (τ_donor_) (Figure 1b). By applying frequency‐domain FLIM (Q2 system, ISS), we analyzed the donor multifrequency data of the “soluble fraction” in Figure 1c and found that the lifetime was better fitted with double‐ rather than single‐exponential decay model. The fitted donor lifetimes (τ_1,_ τ_2_) of 25QHtt, 109QHtt, and 109QHtt treated with 8R10Q were (1.07, 3.51), (0.85, 3.31), (0.94, 3.45), respectively (Figure 1d). The fraction and the fitting details are included in Table S2 (Supporting Information). Comparing with 25QHtt, the donor lifetimes were significantly decreased in 109QHtt. Meanwhile, the addition of 8R10Q increased the fluorescence lifetime of 109QHtt without influencing the lifetime of 25QHtt (Figure 1d; Figure S2 and Tables S2 and S3, Supporting Information). By further applying the ISS software (VistaVision),[qv: 13b] we further derived the phasor plot of the soluble fraction in Figure 1c (Figure S3a, Supporting Information) and calculated the corresponding FRET efficiency (0–100%) from the curved trajectory (details in the “Experimental Section”) (Figure 1e). Pixels highlighted in purple correspond to the phasors within green or red circles. Our results indicated that the FRET efficiency in 109QHtt (8.7%) is much higher than 25QHtt (1.2%), and the addition of 8R10Q decrease its efficiency (4.5%). Quantitative FRET analysis (Figure 1f) agreed well with the aforementioned case. Together, these results suggested that 8R10Q reduced the interactions between soluble 109QHtt to form less‐compact oligomers.

We further characterized the oligomerization state with or without the administration of 8R10Q in 25QHtt‐eYFP and 109QHtt‐eYFP expressing cells by size‐exclusion column (SEC). Each fraction was probed with Huntingtin‐specific antibody, EM48, to monitor the size distribution of Htt‐eYFP protein. Treatment of 8R10Q peptide in 109QHtt‐eYFP expressing cells decreased high‐order oligomers compared with 109QHtt‐eYFP only and 109QHtt‐eYFP treated with scrambled 8R10Q peptide (s8R10Q), indicating that 8R10Q is able to prevent mHtt to form high‐order oligomers (Figure 1g). Taken together from the FLIM–FRET and SEC data as depicted above, it is suggested that 8R10Q shifted the intracellular mHtt oligomerization process by reducing the interactions between individual 109QHtt molecules to form low‐ordered, less‐compact mHtt intermediates.

### 2.2 Inhibition of mHtt Aggregation and Reduction in the Compactness of mHtt Aggregates by 8R10Q in Neuro2a Cell

**Figure 2:**
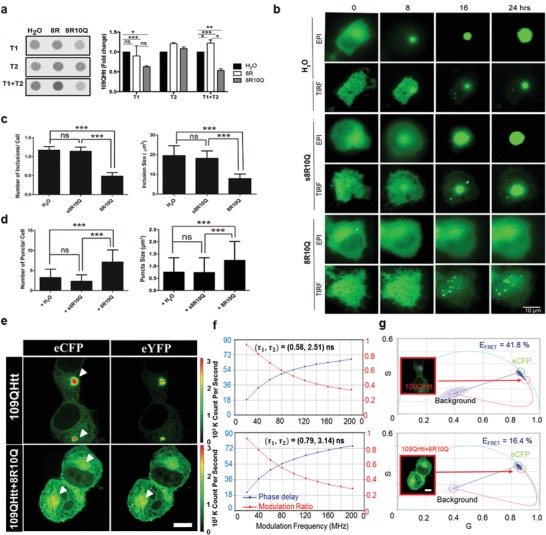
Treatment of 8R10Q prevented large mHtt inclusion formation in Neuro2a cell. a) Left panel: filter trap assay of 109QHtt treated with H_2_O or peptides (8R, 8R10Q) at the 8th hour (*T*1), the 24th hour (*T*2), or treated twice (*T*1+*T*2) after transfection. Right panel: all samples were probed with anti‐Huntingtin antibody, EM48, and further quantified using Image J. b) Epifluorescence and TIRF microscopic images of the living Neuro2a expressing 109QHtt‐eYFP treated with water, s8R10Q, or 8R10Q peptides. Scale bar: 10 µm. c) Quantitation of the number of inclusions per cell and its size in Neuro2a cells 24 h after water or peptide treatment. d) Quantitation of the number of puncta in each cell and its size in Neuro2a cells 24 h after peptide treatment. e) Neuro2a cells co‐expressing 109QHtt‐eCFP and 109QHtt‐eYFP (top) and 109QHtt‐eCFP and 109QHtt‐eYFP in the presence of 8R10Q (bottom). f) Donor multifrequency FLIM data of the aggregated fraction in panel (e) were fitted with the double‐exponential decay model. Additional information was summarized in Table S4. g) The phasor plot of the aggregated fraction in panel (e) and the corresponding FRET efficiency (0–100%) from the curved trajectory. Pixels highlighted in purple correspond to the phasors within green or red circles. All data were shown as mean ± standard deviation (s.d.), *N* = 4 in panel (a); *n* = 35 in panels (c) and (d). Statistics were done with two‐way ANOVA followed by posthoc Tukey's test for panel (a); one‐way ANOVA followed by posthoc Tukey's test for panels (c) and (d). ***p < 0.001; ns: not significant. Scale bar: 10 µm.

The ability of the amphiphilic peptide to modulate mHtt aggregation was further tested in the Neuro2a cell model. As shown in **Figure** 2a, 20 × 10^−6^ m of peptide 8R10Q administered at 8 h (*T*1) or treated twice at 8 and 24 h (*T*1+*T*2) after transfection with 109QHtt‐eYFP construct, significantly reduced 109QHtt‐eYFP aggregation compared with water, or 8R peptide control by filter trap assay. In contrast, 8R10Q failed to inhibit aggregation when administered at 24 h (*T*2). These results showed that 8R10Q peptides effectively prevented mHtt from aggregation at the early stage (Figure 2a, right panel).

To gain detailed insight into the impact 8R10Q in the mHtt aggregation process, we monitored the number and size of the mHtt aggregate population including the large inclusions and small puncta species using epifluorescence and total internal reflection fluorescence (TIRF) microscopy for 24 h. As shown in Figure 2b, large solid inclusions were readily observed in 109QHtt‐eYFP expressing cells treated with water or scrambled peptide (s8R10Q) in comparison with 25QHtt‐eYFP (Figure 2b; Figure S4, Supporting Information). However, small puncta were predominately seen in 109QHtt‐eYFP expressing cells treated with 8R10Q and remained throughout the observed time points (Figure 2b). Quantitative data demonstrated that the average numbers of the inclusions from water, s8R10Q‐treated cells, or 8R10Q‐treated cells were 1.12, 1.14, and 0.49, respectively (Figure 2c, left panel). The average sizes of the inclusions from water, s8R10Q‐treated cells, or 8R10Q‐treated cells were 19.5, 18.1, and 7.85 µm^2^, respectively (Figure 2c, right panel), indicating that administration of 8R10Q significantly reduced the size and the number of the inclusions. In addition, 8R10Q‐treated cells showed the significantly increased number of small puncta to approximately twofolds. The size of puncta compared to water or s8R10Q‐treated cells also increases by 1.5‐folds (Figure 2d). Altogether, these results indicate 8R10Q interfere the 109QHtt aggregation process to form “puncta species” and prevented the formation of “large inclusions.”

While the impact of 8R10Q on the compactness of the soluble mHtt was characterized previously (Figure 1c–g), we also examined the effect of 8R10Q on the compactness of the mHtt inclusions and puncta species here. We analyzed the donor multifrequency data of the “aggregated fraction” in Figure 2e and fitted the lifetime with the double‐exponential decay model. The fitted donor lifetimes (τ_1,_ τ_2_) in 109QHtt and 109QHtt with 8R10Q treatment were (0.58, 2.51) and (0.79, 3.14), respectively (Figure 2f). The fraction and the fitting details are included in Table S4, Supporting Information. Comparing with 25QHtt (Figure 1d), the donor lifetimes were significantly decreased in the large inclusions of 109QHtt (Figure 2f). Meanwhile, the addition of 8R10Q further increased the fluorescence lifetime of 109QHtt. We further derived the phasor plot (Figure S3b, Supporting Information) of the aggregated fraction in Figure 2e and calculated the corresponding FRET efficiency (0–100%) from the curved trajectory (details in the “Experimental Section”) (Figure 2g). Pixels highlighted in purple correspond to the phasors within green or red circles (Figure 2g). Our results indicated that the addition of 8R10Q decreases the FRET efficiency of 109QHtt from 41.8% to 16.4%. Quantitative FRET analysis (details in Figure S5 in the Supporting Information) agreed well with the aforementioned data. Together, these results suggested 8R10Q inhibited “compacted” but allowed “less compacted” aggregation formation.

Inspired by the recently disclosed molecular mechanisms of heterogeneous mHtt oligomerization by Bonfanti et al.,^17^ we further evaluated the impact of 8R10Q on heterogeneous mHtt by applying FLIM measurement and filter trap assay (details in the “Experimental Section”). To generate heterogeneous mHtt, we co‐expressed 109QHtt‐eCFP and 25QHtt‐eYFP in Neuro2a cell. By analyzing the decreased FRET efficiency (Figures S6–S8, Supporting Information) and the decreased EM48‐positive aggregates of the heterogeneous mHtt (Figure S9, Supporting Information) upon the addition of 8R10Q, we demonstrated that 8R10Q maintained heterogeneous mHtt in low‐ordered intermediates, reduced the compactness of insoluble heterogeneous mHtt, and inhibited its inclusion formation.

### 2.3 8R10Q Interfered the Oligomerization and Aggregation Process of polyQ‐Rich Peptide Aggregates as Observed by Frequency‐Domain FLIM

**Figure 3:**
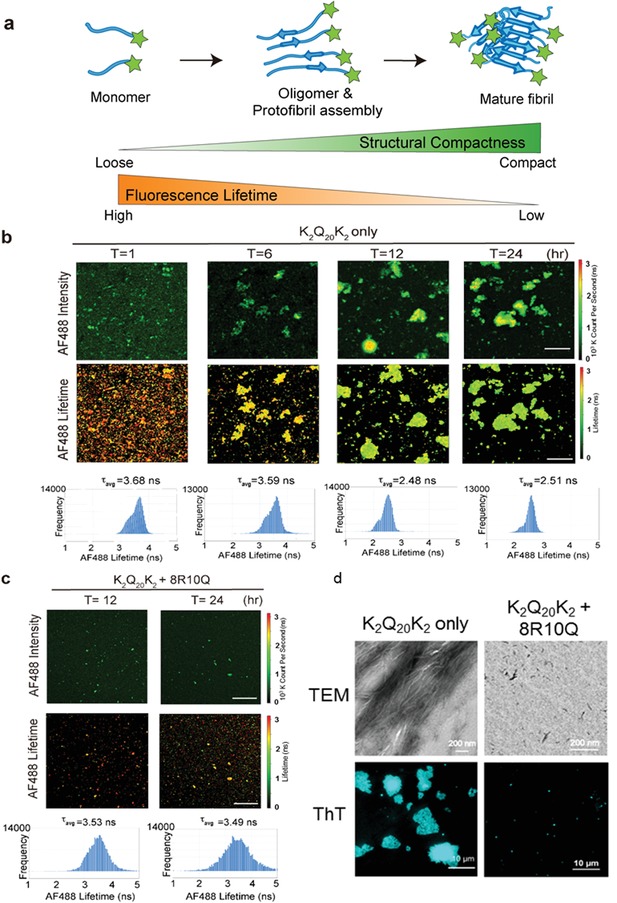
8R10Q reduced the compactness of polyQ‐rich peptide (K_2_Q_20_K_2_) aggregates. a) Illustration of the fluorescence‐labeled polyQ‐rich peptide forms into oligomers, protofibrils, and mature fibrils during the oligomerization and aggregation process. The structural compactness of these assemblies increases during the aggregation process (green color), accompanied by the decrease in the fluorescence lifetime (orange color). b) Representative image of K_2_Q_20_K_2_ probed with polyQ‐specific antibody, 1C2, followed by Alexa Fluor 488 secondary antibody (AF488) and acquired by fluorescence lifetime imaging microscopy (FLIM). Fluorescence of AF488 (upper lane), its lifetime images (middle lane), and the corresponding AF488 fluorescence lifetime frequency histograms (lower lane) were taken at the indicated time points (*T* = 1, 6, 12, 24). The color coding of AF488 intensity and lifetime corresponds to the color bar on the right side. Note the AF488 fluorescence lifetime decreased during K_2_Q_20_K_2_ aggregation from *T* = 1 to *T* = 24, indicating a transition of the K_2_Q_20_K_2_ population toward the compact fibril state. Scale bar: 10 µm. c) K_2_Q_20_K_2_ treated with 8R10Q peptide and acquired image by FLIM. Representative fluorescence of AF488 (upper lane), its lifetime images (middle lane), and the corresponding fluorescence lifetime frequency histograms (lower lane) taken at the 12th and 24th hours. Scale bar: 10 µm. d) The TEM (upper panel) and ThT staining (lower panel) images of K_2_Q_20_K_2_ in the presence and absence of 8R10Q. Images were taken after 24 h incubation. Scale bar on the upper lane: 200 nm; bottom lane: 10 µm.

To assess the mechanism by which 8R10Q interfered the oligomerization and aggregation process of polyQ‐expanded mHtt, we further applied synthetic polyQ‐rich peptide (K_2_Q_20_K_2_), a useful model system which has been applied to investigate the polyQ‐mediated aggregation in huntingtin, ataxins, and other proteins associated with the trinucleotide expansion disease.^18^ Here, frequency‐domain FLIM was applied to track the structural compactness of K_2_Q_20_K_2_ upon aggregation. K_2_Q_20_K_2_ was first detected by the polyQ‐specific antibody 1C2 and then visualized by the Alexa Fluor 488 (AF488)‐coupled secondary antibody. The AF488 fluorescence lifetime was measured in the presence/absence of 8R10Q, and a decrease in the lifetime directly correlated with the compactness of K_2_Q_20_K_2_ in the aggregate species due to the self‐quenching of the neighboring fluorophores (**Figure** 3a). Our results showed that aggregate species with increased AF488 photon counts were indeed observed (Figure 3b, upper row). The average lifetime of the monomeric K_2_Q_20_K_2_ was 3.87 ns, which was determined by measuring the AF488‐coupled secondary antibody only (data not shown). During aggregation, the average of the AF488 fluorescence lifetime decreased from 3.68 ns at the 1st hour to 2.51 ns at the 24th hour (Figure 3b, middle and lower rows), indicating that K_2_Q_20_K_2_ gradually assembled into large and compact aggregate clusters with time. Consistent with our data in the cellular model (Figure 2), only small puncta were observed in the K_2_Q_20_K_2_ samples treated with 8R10Q at the 12th and 24th hours (Figure 3c, upper row). FLIM results also showed that the average fluorescence lifetime of these puncta was longer than the K_2_Q_20_K_2_ only group which retained mostly at 3.49 ns at the 24th hour (Figure 3c, middle and lower rows). These results indicated that 8R10Q was able to halt the aggregation process at structurally loose puncta species.

To further characterize the impact of 8R10Q on the amyloid and structural property of K_2_Q_20_K_2_ aggregates, we applied ThT (a fluorescence dye which binds to amyloid fibril) staining, TIRF microscopy and TEM. As shown in TEM imaging, K_2_Q_20_K_2_ formed short nanofibrils after 12 h, which subsequently assembled into highly ordered fibrils in bundles after 24 h (Figure 3d; Figure S10a, Supporting Information). The treatment of 8R10Q significantly reduced the fiber formation of K_2_Q_20_K_2_ after 24 h (Figure 3d). In addition, K_2_Q_20_K_2_ formed ThT‐positive puncta after 1 h incubation, grew small clusters after 6 h, and finally formed large aggregates after 24 h under TIRF microscopy (Figure 3d; Figure S10b, Supporting Information). By contrast, 8R10Q inhibited formation of ThT‐positive aggregates (Figure 3d; Figure S10b, Supporting Information). We thus demonstrated that 8R10Q was able to halt the fibrillization process and suppress amyloid formation of K_2_Q_20_K_2_.

### 2.4 8R10Q Protected Cells against mHtt Neurotoxicity

We further evaluated the biological effect of 8R10Q in mHtt‐expressing cell. 25QHtt‐expressing cells showed ≈83% of survival in all groups (Figure S11a, top graph, Supporting Information). In 109QHtt‐expressing Neuro2a cell, the cell survival decreased to ≈72%. However, administration of 8R10Q increased the survival back to 80% (Figure S11a, bottom graph, Supporting Information). In addition, undifferentiated Neuro2a cells expressing 25QHtt or 109QHtt were subjected with retinoic acid (RA) to induce differentiation into neurite‐bearing cells. The 109QHtt group decreased the number of cells bearing RA‐induced neurites compared with the 25QHtt group (Figure S11b,c, Supporting Information). 8R10Q treatment significantly reversed the defect in neurite outgrowth caused by 109QHtt compared with water or scrambled control. These results clearly showed that 8R10Q could reduce 109QHtt‐induced neurotoxicity and exerted neuroprotective effect.

### 2.5 8R10Q Specifically Localized in the Brain and Ameliorated mHtt‐Induced Functional Deterioration in Mice

**Figure 4:**
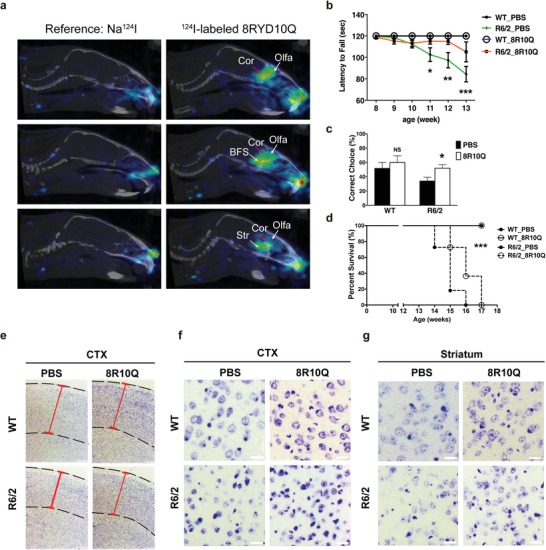
8R10Q ameliorated functional deterioration and neuronal damage of R6/2 transgenic mice. a) PET/CT images for ICR mice administered with ^124^I‐8RYD10Q peptide and Na^124^I through intranasal administration. Arrowheads indicate the uptake and biodisposition of ^124^I‐8RYD10Q peptide in various regions of brain, predominantly in the olfactory bulb (Olfa), cortex (Cor), basal forebrain of the septal area (BFS), and striatum (Str) in addition to nasal cavities. The free ionic iodine‐124 (from Na^124^I) cannot be observed in the brain using the same imaging protocol, data processing, and image presentation method. *N* = 3 in both Na^124^I and ^124^I‐8RYD10Q administered group. b) Longitudinal Rotarod performance of wild‐type (WT) and R6/2 mice treated with PBS or 8R10Q peptide. c) T‐maze test of WT and R6/2 mice at 13 weeks of age. Administration of 8R10Q peptide significantly rescued the memory deficit in R6/2 mice. d) Lifespan of WT R6/2 mice treated with PBS or 8R10Q. 100% of R6/2 mice died before 16 weeks of age. 8R10Q significantly extended the lifespan of R6/2 mice. e) Microscopic images of the Nissl stain sections of cortex (CTX) of WT and R6/2 mice. The cortex and its thickness were highlighted by the dashed lines and red solid lines, respectively. Note the decrease in cortical thickness in R6/2 mice, which was reversed by the 8R10Q peptide. f,g) Microscopic images represent the Nissl stain sections of cortex (CTX) and striatum in WT and R6/2 mice. Note the decrease in neuron numbers in cortex and striatum was significantly reversed by 8R10Q. *N* = 3 per group; each bar was the average of five sections. All data were shown as mean ± s.d.; WT mice *N* = 6 per group; R6/2 mice *N* = 10 per group. Statistics done by two‐way ANOVA: b,c), *p < 0.05; **p < 0.01; ***p < 0.001, ns: not significant, Log‐rank (Mantel‐Cox) Test d), ***p < 0.0001.

To investigate the capability of 8R10Q being delivered into brain, the peptide labeled with iodine‐124 (^124^I‐8RYD10Q) was intranasally administered into mice. The positron emission tomography (PET) imaging was carried out to detect the intracerebral biodisposition of ^124^I‐8RYD10Q compared to ionic Na^124^I as control group (details in the “Experimental Section”). The presence of ^124^I‐8RYD10Q in mice brains was observed, and the olfactory bulb (Olfa), cortex (Cor), basal forebrain of the septal area (BFS), and striatum (Str) regions were highlighted (**Figure** 4a). In contrast, no signal could be observed in brains of mice receiving intranasal Na^124^I treatment. These results confirmed that intranasal administered 8R10Q peptide could be delivered into mouse brains.

To test the therapeutic effect of the peptide in vivo, the 8R10Q peptide was delivered through intranasal aspiration 6 days/week into the wild‐type (WT) or R6/2 transgenic mice, the HD animal model expressing Htt exon 1 with an increased CAG repeat expansion, when they were 4 weeks of age. The body weights of the WT or R6/2 mice treated with 8R10Q through intranasal delivery remained comparable to those treated with phosphate‐buffered saline (PBS). As shown in Figure 4b, the motor deterioration assessed by the Rotarod of R6/2 mice became significant from 11 weeks of age, but the 8R10Q treatment effectively delayed the phenotype until 13 weeks. In addition, 8R10Q also significantly corrected the memory deficit of 13 weeks old R6/2 mice as shown in the T‐maze test (Figure 4c). Lastly, all R6/2 mice treated with PBS died before or at 16 weeks of age; however, 8R10Q given intranasally extended survival to 41% at 16 weeks of age (Figure 4d). To sum up, the 8R10Q effectively delayed the onset of disease and ameliorated the phenotypes.

### 2.6 8R10Q Ameliorated Neuronal Damage and mHtt Aggregation in R6/2 Mice

R6/2 transgenic mice exhibited a decrease in the cortical thickness due to neurodegeneration. Interestingly, 8R10Q peptide prevented the cortical thinning (Figure 4e; Figure S12a, Supporting Information), suggestive of its ability to rescue neurons from death. Indeed, the neuronal count revealed that loss of neurons in both cortex (Figure 4f; Figure S12b, Supporting Information) and striatum (Figure 4g; Figure S12c, Supporting Information) of the R6/2 mice was significantly prevented by intranasal 8R10Q treatment.

To correlate the beneficial effect with the aggregates of mHtt, we conducted the immuno‐histochemistry to calculate the areas occupied by the aggregates per unit area (150 µm^2^). As shown in Figure S12d–h (Supporting Information), 8R10Q peptide decreased the areas of aggregates by ≈60% in both cortex and striatum compared with PBS control. In addition, the intensity which represented the semiquantitative amount of mHtt in the aggregates was also significantly decreased by 8R10Q peptide in both areas. Astrocytosis and microgliosis are well‐established responses, which accompany with neurodegeneration and contribute to the pathogenesis of disease. Along with the rescue, effect in neuronal loss and mHtt aggregation was the significant decrease in the immunoreactivity of glial fibrillary acidic protein (GFAP)‐positive astrocytes (Figure S12i, Supporting Information) and that of Iba‐1 positive microglia (Figure S12j, Supporting Information) in the cortex of the 8R10Q‐treated R6/2 mice. Taken together, these results showed that 8R10Q effectively decreased the neurotoxicity induced by the mHtt in vivo.

## 3 Discussion

To identify peptides that will decrease mHtt toxicity, studies using various approaches such as phage display[qv: 5c,19] or other screening methods^20^ and sequence modifications^21^ have previously been conducted. Modularity is one of the fundamental principles at all levels of organizations in biology.^22^ For instance, a protein may be considered a multidomain platform built on the modularity principle to control amazingly diverse and complex processes with precise temporal and spatial control through interactions of each domain with it diverse binding partners.^23^ Our amphiphilic octaarginine‐based peptides were designed by this principle. Compared with previous peptide approaches, our design of amphiphilic peptide showed at least three advantages: 1) the ability to tackle mHtt at the early stage of aggregation process, decreased compact mHtt oligomer/aggregate formation by the repulsion force, which resulted in neuroprotection, and therapeutic effect in the cellular and HD mice model. 2) While mHtt is an intracellular protein, our design to integrate polyR sequence into the amphiphilic peptide played a critical role to both enhance the cell permeability and provide a repulsion force for intracellular aggregation. 3) The dual‐functions peptide showed the possibility of extending the design of amphiphilic peptides against other neurodegenerative diseases by choosing the partner sequence with specific affinity for the misfolded protein/derivative of the disease of interest. A separate study applying the similar principle also yielded beneficial outcome in APP/PS1 transgenic mice, a widely used mouse model for AD.^24^ This study provides clear evidence for the possibility of extending this design to other neurodegenerative diseases characterized by misfolded proteins, which is especially significant for a large number of the diseases that received less research attention due to low incidence.

The oligomerization and aggregation process of mHtt protein is believed to be one of the critical pathological processes to cause neuronal dysfunction and death in Huntington's disease.^25^ To characterize the molecular events during the mHtt aggregation process as well as directly track the transient and subtle changes in protein conformational dynamics in vitro and in cell still remained challenging. Various intensity‐based fluorescent methods have been employed to disclose the morphological and structural property of amyloid oligomer or aggregate directly including super‐resolution fluorescence microscopy, FRET, and fluorescence correlation spectroscopy (FCS) with photon counting histogram.[qv: 12c] The lifetime‐based fluorescence observation is less prone to be affected by fluorophore concentration, method of measurement, fluorescence intensity, photobleaching, and excitation intensity, as it is an intrinsic property of a fluorophore. In this study, we applied FLIM individually based on self‐quenching of dyes and energy transfer between fluorophores to interrogate the structural compactness of oligomers/aggregates of K_2_Q_20_K_2_ and mHtt upon 8R10Q treatment in vitro and in cell. Our results indicate that 1) FLIM is able to capture the dynamic conformational change of polyQ or intracellular mHtt aggregation; and 2) polyQ or mHtt aggregate self‐assembled into compact aggregate structure. Our strategy of applying 8R10Q against mHtt reduced the inter‐ and intramolecular interactions between individual 109QHtt molecules to form less‐compact mHtt intermediates, which assembled into structurally loose small puncta in cell. In fact, Chen et al. recently monitored the aggregation of Tau protein by analyzing the decreased fluorescence lifetime originated from the self‐quenching effect on the protein‐attached fluorophores.^26^ The demonstration of applying FLIM in vitro and cellular system is shown to be a powerful tool to monitor the structural details during biological self‐assembly, and such information can further provide linkage in cellular phenotypes or therapeutic efficacy in future drug design.

Currently, various of peptide‐based inhibitors have been developed against protein misfolding neurodegenerative disease.^27^ QBP1, a well‐known synthetic peptide that has been identified through phage display screening, showed strong ability to prevent the misfolding and aggregation of the expanded polyQ protein in the cell and *Drosophila* model.^28^ However, administration of this peptide drug showed limited therapeutic effect in R6/2 mice due to low drug delivery efficiency in targeting the brain.^29^ Previous study has identified the application of the cell‐penetrating peptide, for example, the arginine‐rich sequence showed strong penetrating ability in the brain of Aβ transgenic mice^30^ or adult zebrafish with low cytotoxic effect upon administration.^31^ In our study, intranasal administration of the amphiphilic octaarginine‐based peptide showed dominant signals in the olfactory bulb, cortex, basal forebrain of the septal area, and striatum regions in addition to nostril and nasal cavity in the PET imaging study, indicating the ability of 8R10Q to transport into brain through olfactory bulb. In addition, we also demonstrated the feasibility of specifically delivering the amphiphilic peptide into the brain through intranasal route. The administration of 8R10Q effectively ameliorated mHtt‐induced functional deterioration and neuronal damage in R6/2 mice. In summary, a new strategy has been presented in tackling mHtt oligomers and aggregates by harnessing amphiphilic octaarginine‐based peptide through intranasal delivery, which may shed light on the development of novel chemical engineering in the biomedical field.

## 4 Experimental Section

*Plasmid Constructs*: cDNA encoding Htt exon 1 fragments containing different lengths of polyglutamine tracts (Q_25_ and Q_109_) were subcloned from pcDNA3.1‐Htt‐(Q)_25_‐hrGFP and pcDNA3.1‐Htt‐(Q)_109_‐hrGFP constructs into pcDNA3 vector (Invitrogen Life Technologies). eCFP and eYFP were subsequently fused at the C‐terminus of each construct. All constructs were confirmed by DNA sequencing. Only eCFP and eYFP constructs were used as control plasmids, respectively.

*Peptide Preparation and Identification*: All peptides (8R, 8R10Q, s8R10Q, and K_2_Q_20_K_2_) were synthesized by the batch fluorenylmethoxycarbonyl (FMOC) polyamide method on the peptide synthesizer (PS3, Rainin Instrument, USA). Rink amide AM resin was selected as the solid support. After cleaved from resin, crude peptides were purified by the high‐performance liquid chromatography (HPLC) (Waters 2690, Waters Corp., USA) with reverse phase semipreparative column (C18). The gradient separation was achieved by mixing Buffer A (5% acetonitrile/0.1% TFA/94.9% water) and Buffer B (0.1% trifluoroacetic acid (TFA)/99.9% acetonitrile). The flow rate was kept at 3 mL min^−1^. Peptides purity were confirmed by reversed phase high performance liquid chromatography (RP‐HPLC) with analytical column (C18) and identified by matrix‐assisted laser desorption/ionization (MALDI) (Applied Biosystem, USA) mass spectroscopy.

*Transmission Election Microscopy*: 8R10Q (0.5 × 10^−6^ m), K_2_Q_20_K_2_ only (50 × 10^−6^ m), K_2_Q_20_K_2_+8R10Q (50 × 10^−6^ m) groups were prepared in PBS buffer (14 × 10^−3^ m KCl, 10 × 10^−3^ m sodium phosphate, pH = 7.5), followed by incubation at 37 °C for 24 h. The resulting aliquots of peptide solutions (5 µL) were applied on glow‐charged 300 mesh formvar‐ and carbon‐coated copper grids stained with 1% uranyl acetate. After drying overnight, all samples were analyzed using a JEM‐2011 electron microscope (JEOL, Japan).

*Dynamic Light Scattering*: The particle size was calculated from the Brownian motion of the articles using the Stokes–Einstein equation. The method yields a hydrodynamic diameter, which is a calculated particle diameter of a sphere that has the same measured motion in the solute as the actual particle. 8R10Q (5 × 10^−6^ m) was prepared in H_2_O. The size of the 8R10Q vesicle was measured using DLS with zetasizer (Malvern Zetasizer Nano ZS, Malvern Instruments, Worcestershire, UK). The analysis was performed with a He–Ne laser (633 nm) at a scattering angle of 175° at 25 °C. Size measurements were done in triplicate for each sample.

*Time‐Lapse TIRF Imaging for In Vitro Amyloidogenesis*: Time‐lapse TIRF image collection was carried out using a Nikon TiE microscope, where samples were illuminated with a 405 nm laser light source for the ThT excitation. For ThT staining, 6 × 10^−6^ m of K_2_Q_20_K_2_ only, K_2_Q_20_K_2_+8R10Q, and K_2_Q_20_K_2_+8R15Q were prepared in 10 × 10^−3^ m HCl and 10% hexafluoroisopropanol (HFIP) (v/v) mixed with the same amount of ThT (200 × 10^−6^ m in PBS buffer) solution. An aliquot of 200 µL was mounted on a 35 mm glass bottom dish (Ibidi, Germany) and was cap‐sealed to prevent the loss of the liquid. Images were acquired by time‐lapse TIRF microscopy with a 405 nm laser. The ThT signals were filtered with an eCFP cube (Chroma) and collected by an Andor iXon3 888 back‐illuminated high‐sensitivity electron multiplying charge‐coupled device (EMCCD) camera.

*Time‐Course Epifluorescence and Total Internal Reflection Fluorescence Microscopy*: Neuro2a cells were cultured in Dulbecco's modified Eagle's medium (DMEM; Gibco) containing 2 × 10^−3^ m glutamine, 10% heat inactivated fetal bovine serum, and 100U U mL^−1^ penicillin–streptomycin liquid (Gibco) at 37 °C in a humidified atmosphere containing 5% CO_2_. 5 × 10^4^ of Neuro2a cells in sterile 35 mm µ‐Dish (ibidi, Martinsried, Germany) were transfected with 1.5 µg of 109QHttGFP plasmid by TurboFect Transfection Reagent (Thermo Scientific) according to manufacturer's recommendations. After 8 h of transfection, 109QHttGFP transfected cells were followed by exposure to amphiphilic peptides. TIRF microscopy utilizes evanescent wave to selectively measure the cell surface and matrix areas of approximately several hundred nanometers in depth. Time‐course epifluorescence or TIRF image collection was carried out using a NIKON TiE microscope where samples were illuminated with an ultrahigh pressure 130 W mercury lamp for UV excitation or a 488 nm laser light source. Filters were used to collect fluorescence emission including excited eGFP (excitation D480/40, dichroic D505LP, emission D535/50) and carboxytetramethylrhodamine (TAMRA) (excitation D535/50, dichroic D565LP, emission D590LP) cubes. Cellular images were captured with an Andor iXon3 888 back‐illuminated high‐sensitivity EMCCD camera. Images were analyzed and quantified using Nikon NIS element software.

*Frequency‐Domain Fluorescence Lifetime Imaging*: We applied Q2 FastFLIM system from ISS company (https://www.iss.com/) and Nikon Ti‐U inverted microscope with sub‐micrometer automatic controlled *XYZ* stage (detailed optical configuration is shown in Figure S13 in the Supporting Information). A temperature‐controlled sample chamber with an irrigation system was used for long‐time FLIM measurements in living cells. A *XY* set of Galvo mirrors was used for nanoposition control of the FLIM image. An oil‐immersion objective (Nikon Plan Apo 100×/numerical aperture (NA) 1.4) mounted on a piezodevice was applied. The system equipped with 440 nm (4 mW) and 488 nm (5 mW) sub‐nanosecond modulated pulsed laser at the fundamental frequency of 20 MHz was controlled by ISS VistaVision software, which was used for CFP and YFP excitation sources. An EM1 filter (530/43 nm bandpass filter) was used for Channel 1 GaAs photomultiplier tube (PMT) detector and EM2 filter (480/40 nm bandpass filter) was used for Channel 2 GaAs PMT detector (Hamamatsu, H7422P‐40). To avoid strong fluorescence from 109QHtt, the laser power used for the FLIM measurements was under 30% to achieve a reasonable photon count rate and avoid possible pile‐up effect (e.g., Figures 1 and 2, Figures S14–S16 and Tables S5 and S6, Supporting Information).[qv: 13a] On the other hand, the laser power for 25QHtt was around 50%. Calibration of the system and phasor plot space was performed by measuring fluorescein in ddH_2_O, which has a known single exponential lifetime of 4.04 ns (Figure S14, Supporting Information).^32^

*FLIM FRET Data Analysis*: The phase delays and modulation ratios of the emission relative to the excitation were measured at multifrequency modulation frequencies for each pixel of an image. The measured phase delays and modulation ratios were analyzed by the ISS VistaVision software to extract the lifetime information. The VistaVision software allows both the fitting and phasor plot analysis for data analysis and calculates the standard weighted least squares (χ^2^) as a quantitative measurement of the fitting significance. The phasor data analysis was performed using the ISS VistaVision as described in previously published papers.[qv: 13b,32] In brief, each pixel of the FLIM data gives rise to a phasor in the phasor plot. A threshold was applied to eliminate background noise, and a Gaussian 2D spatial convolution filter was used for smoothing. Since phasors follow vector algebra, it is possible to determine the fractional contribution of two or more independent molecular species coexisting in the same pixel. In the FRET experiment, the lifetime of the donor molecule is changed upon interaction with an acceptor molecule. The realizations of all possible phasors that are quenched with different efficiencies describe a curved FRET trajectory in the phasor plot. The experimental position of the phasor of a given pixel along the trajectory determines the amount of quenching and therefore the FRET efficiency. The contributions from the background (autofluorescence from cell only group) and the donor without acceptor (eCFP alone group in Figure S15b in the Supporting Information) were evaluated using the rule of the linear combination, with the background phasor and the donor unquenched being determined independently.

*Filter‐Trap Analysis*: 30 µg of total protein radioimmunoprecipitation assay (RIPA) lysate per sample was loaded onto prerinsed 0.2 µm nitrocellulose membrane (GE Healthcare) sandwiched in the Dot Blotter Unit (Major Science, Saratoga, CA, USA). After vacuum suction applied, the membrane was removed and briefly rinsed in RIPA buffer. Samples were probed with either EM48 or β‐actin antibody, and quantified by Image J (right panel in Figure S14, Supporting Information).

*Animal and Peptide Treatment*: R6/2 mice and littermate controls were obtained from Jackson Laboratory (Bar Harbor, ME, USA), and mated to female control mice (B6CBAFI/J). Offspring was genotyped by a polymerase chain reaction (PCR) with tail genomic DNA using primers (5′‐CCGCTCAGGTTCTGCTTTTA‐3′ and 5′‐GGCTGAGGAAGCTGAGGAG‐3′). The number of CAG repeats of R6/2 mice used in the present studies was between 194 and 203. Mice were housed at the Institute of Biomedical Sciences Animal Care Facility (Taipei, Taiwan) under a 12 h light/dark cycle. Animal experiments were performed under protocols approved by the Academia Sinica Institutional Animal Care and Utilization Committee (Taipei, Taiwan). The indicated peptide was dissolved in H_2_O to a final concentration of 2 mg mL^−1^. Treatment was started when a mouse reached 4 weeks of age by applying 2.5 µL of 2 mg mL^−1^ peptide (1.7 nmol) to each nostril 6 days a week and continued through 13 weeks of age. For survival study, the treatment was stopped at 17 weeks of age. Animal experiments were done with 6 WT mice and 10 R6/2 mice. The statistical analyses were conducted with one‐way analysis of variance (ANOVA).

*Rotarod Assay*: Motor coordination was assessed with a rotarod apparatus (model 7600; UGO BASILE, Comerio, Italy) at a constant speed of 12 rpm over a period of 2 min essentially as previously described. All mice were tested three times per week. For each test, animals were placed in the apparatus before the initiation of rotation. Each mouse was given three trials for a maximum of 2 min for each trial. The statistical significance was analyzed by one‐way ANOVA.

*T Maze Assay*: To assess the cognitive function, each mouse was subjected to the T maze assay as described previously.^33^ Briefly, each mouse at the start area was allowed to walk to the intersection where the right or left arm was closed. The animal was subjected to the maze again. By nature, it would explore the other arm which was not visited in the previous trial. Each animal was tested for ten consecutive trials. The number of alternative choices out of ten trials was recorded and presented as the percentage of correct choices. The statistics was done with the Student‐*t* test.

*Cortical Thickness and Neuron Count*: Nissl‐stained 5 µm thick brain sections of the corresponding neocortex and neostriatum were used for this purpose. Three consecutive sections of one mouse were used, and each experimental group included five different mice. The images were captured by an RT3 digital camera, and analyzed with Metamorph software (Molecular Devices, Sunnyvale, CA, USA). Statistics was conducted with the Student‐*t* test.

*Immunostaining of Brain*: 5 µm thick paraffin‐embedded brain sections were deparaffinized, rehydrated, blocked with 5% hydrogen peroxide/methanol, antigen‐retrieved with 1× 10^−3^ m ethylenediaminetetraacetic acid (EDTA) (pH 8), and then stained with EM48 (1:500, Chemicon International, Temecula, CA) or Iba‐1 (1:500, Novus Biologicals, Littleton, CO, USA) monoclonal antibody and peroxidase‐conjugated secondary antibody for immuno‐histochemistry with diaminobenzidine (DAB) or GFAP (1:500, Chemicon) with DyLight 488‐coupled secondary antibody (Jackson ImmunoResearch (West Grove, PA, USA). Images were captured with the RT3 camera and analyzed with the Metamorph software.

*Animals for Intranasal Administration of Radioactive Probe*: Mice (male ICR, 6–8 week old) were purchased from BioLASCO (Taipei, Taiwan) and acclimated for 2 weeks in the Laboratorial Animal Center of the National Health Research Institutes. Mice were raised in the individual ventilation cage by ad libitum feeding of chow and water with a temperature of 24 ± 2 °C and a relative humidity level of 50 ± 10%. Animal protocols were reviewed and approved by the Institutional Animal Care and Use Committee at NHRI (NHRI‐IACUC‐104058‐A).

*Preparation and Administration of Radioactive Peptide*: The 8RYD10Q peptide labeled with radioactive iodine (Na^124^I, PerkinElmer Inc., Waltham, MA) was performed in the precoated iodination tube (Thermo Fisher Scientific Inc., Rockford, lL). In the sequence of 8RYD10Q peptide, tyrosine (Y) was used here for radioactive iodine‐124 labeling reaction. Tris‐iodination buffer (1.0 mL, containing 25 × 10^−3^ m tris‐HCl, pH 7.5, 0.4 m NaCl) was added to a prewet tube and then decant at 1 min later. The Na^124^I (3.0 mCi) was subsequently added into the precoated iodination tube and incubated for activation at room temperature for 6 min. The activated solution was transferred to a vial and mixed with 550 µg of 8RYD10Q peptide in 100 µL of tris‐buffer at 4 °C for 6 min. Radiochemical purity was determined by instant thin layer chromatography (ITLC) using chromatography paper (4 Chr, GE Healthcare UK Limited) as the stationary phase and normal saline (0.9% NaCl) as the mobile phase. Acceptance of radiochemical purity is 95% for quality control of the PET tracer for animal study. For PET imaging, 0.475 mCi of ^124^I‐8RYD10Q peptide in 50 µL solution was delivered into both nostrils of each mouse through intranasal administration. Aliqout of ^124^I‐8RYD10Q peptide or Na^124^I (2.5 µL) was applied in each nostril for ten times.

*PET/CT Imaging*: PET imaging and computed tomography (CT) imaging were performed using a preclinical trimodality imaging system (FLEX *Triumph*; Gamma Medica‐Ideas, Northridge, CA, USA). The X‐ray tube of CT was operated at a voltage of 70 kVp, a current of 0.185 mA, and a geometrical magnification of 3. The 512 projections of CT data were reconstructed by filtered back projection (FBP) with a matrix of 512 × 512 × 512, presenting a pixel size of 80 µm. As for PET imaging, a 60 min dynamic scan of the brain was acquired for 1 h after intranasal injection of ^124^I‐8RYD10Q peptide or Na^124^I followed by a CT scan for anatomic co‐registration. PET images were reconstructed by a maximum likelihood estimation method (MLEM) as a matrix of 92 × 92 × 31 with a pixel size of 0.5 × 0.5 × 1.175 mm. These PET images were subsequently processed by PMOD (version 3.7, PMOD Technologies Ltd, Switzerland) to depict brain region contour. Each of PET images was resliced into a matrix of 512 × 512 × 512 with CT co‐registration. The Altas template for PET images was employed to determine the uptake and biodisposition of ^124^I‐8RYD10Q peptide in various regions of brain.

## Conflict of Interest

The authors declare no conflict of interest.

## Author Contributions

J.J.‐T.H., P.‐H.T., R.‐Y.H., J.‐K.C., and X.‐M.L. made experimental design; R.‐Y.H. and X.‐M.L. performed the cellular experiments; R.‐Y.H. and T.‐S.K. performed the biochemistry experiments; X.‐M.L., Y.‐S.J., and W.‐N.L. performed animal experiments; R.‐Y.H., X.‐M.L., T.‐S.K., Y.‐S.J., and W.‐N.L. analyzed the data; R.‐Y.H., P.‐H.T., and J.J.‐T.H. wrote the manuscript; and P.‐H.T., J.J.‐T.H., Y.C., and R.‐Y.H. gave manuscript guidance.

## Supporting information

Supporting Information
