# Supplementary material for: Nanoscopic Insights of Amphiphilic Peptide against the Oligomer Assembly Process to Treat Huntington's Disease
Source: Adv Sci (Weinh). 2019 Dec 9;7(2):1901165. doi: 10.1002/advs.201901165 (PMC6974936; doi:10.1002/advs.201901165)
Supplement: Supplementary file 1 — Supporting Information [file ADVS-7-1901165-s001.pdf]

Copyright WILEY-VCH Verlag GmbH & Co. KGaA, 69469 Weinheim, Germany, 2019.

Supporting Information

for *Adv. Sci.,* DOI: 10.1002/advs.201901165

Nanoscopic Insights of Amphiphilic Peptide against the Oligomer Assembly Process to Treat Huntington's Disease

Ruei-Yu He, Xiang-Me Lai, Chia-Sui Sun, Te-Shien Kung, Jhu-Ying Hong, Yu-Song Jheng, Wei-Neng Liao, Jen-Kun Chen, Yung-Feng Liao, Pang-Hsien Tu,* and Joseph Jen-Tse Huang**

Supplementary Information

Nanoscopic insights of amphiphilic peptide against oligomer assembly process to treat Huntington's disease

Ruei-Yu He[1,*], Xiang-Me Lai[2,3], Chia-Sui Sun[1], Te-Shien Kung[1,4], Jhu-Ying Hong[1], Yu-Song Jheng[2], Wei-Neng Liao[5], Jen-Kun Chen[3,5], Yung-Feng Liao[6], Pang-Hsien Tu[2,3,*] and Joseph Jen-Tse Huang[1,*]

[1] Institute of Chemistry and [2] Institute of Biomedical Sciences, Academia Sinica, Taipei, Taiwan
[3] Division of Urology, Department of Surgery, Tri-Service General Hospital, National Defense Medical Center, Taipei, Taiwan
[4] National Taiwan University of Science and Technology, Taipei, Taiwan
[5] Institute of Biomedical Engineering and Nanomedicine, National Health Research Institutes, Miaoli, Taiwan.
[6] Institute of Cellular and Organismic Biology, Academia Sinica, Taipei, Taiwan

*Corresponding authors:

1. Joseph Jen-Tse Huang, PhD
   Institute of Chemistry, Academia Sinica
   128 Sec.2 Academia Road, Nankang District, Taipei, Taiwan 11529
   Email: jthuang@gate.sinica.edu.tw
2. Pang-Hsien Tu, MD, PhD
   Institute of Biomedical Sciences, Academia Sinica
   128 Sec.2 Academia Road, Nankang District, Taipei, Taiwan 11529
   Email: benrosetu@yahoo.com
3. Ruei-Yu He, PhD
   Institute of Chemistry, Academia Sinica
   128 Sec.2 Academia Road, Nankang District, Taipei, Taiwan 11529
   Email: imreyu@gmail.com

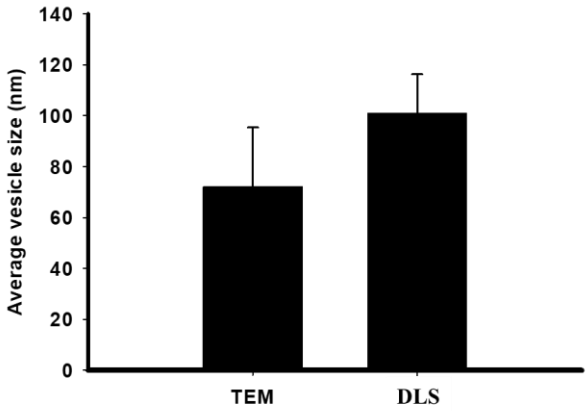

Supplementary Figure 1. Average size distribution of self-assembled vesicles from 8R10Q. The vesicle size was measured by applying TEM and dynamic laser light scattering (DLS), respectively.

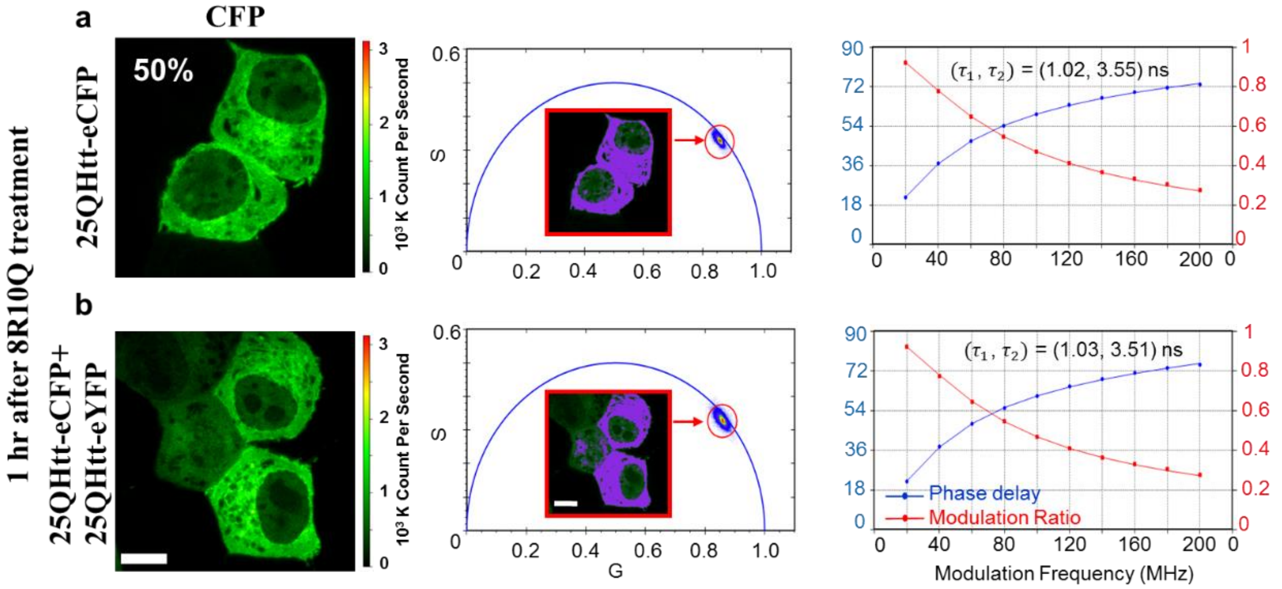

Supplementary Figure 2. Frequency-domain FLIM measurements of Neuro2a cells at 1 hr after 8R10Q treatment. Fluorescence intensity images (512 × 512 pixel, left column), phasor plot analysis (middle column), and multi-frequency lifetime fitting curves (right column) of living Neuro2a cells (a) expressing 25QHtt-eCFP or (b) co-expressing 25QHtt-eCFP and 25QHtt-eYFP measured by frequency-domain FLIM with 440 nm laser of different power (30%, 50% and 70% of laser power) at 20 MHz. For the phasor plot analysis, pixels highlighted in purple correspond to the points selected within the red circle in each phasor plot. Donor multi-frequency FLIM data was fitted by double exponential decay model. Additional information was summarized in Supplementary Table 2. Scale bar: 10 μm.

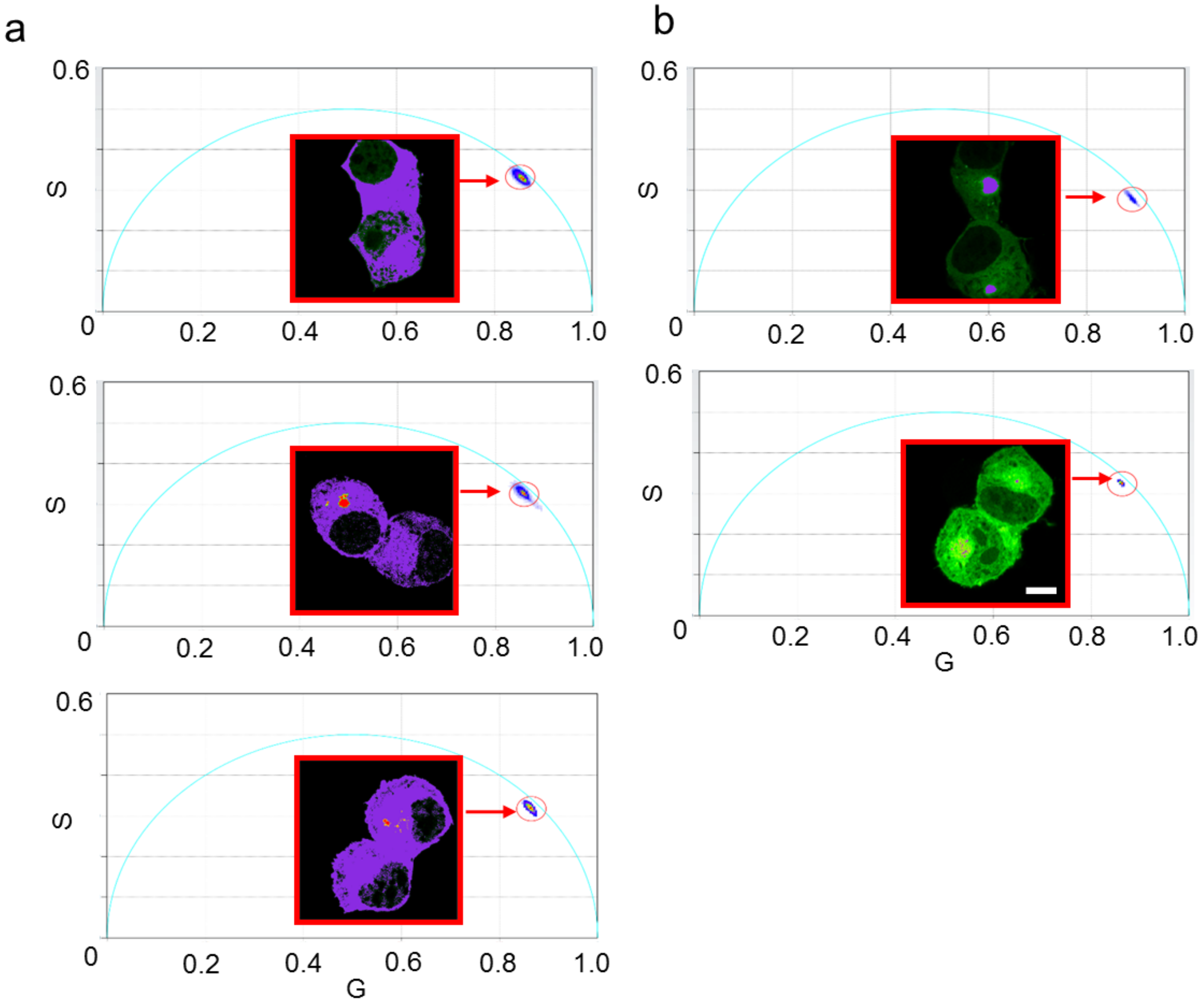

Supplementary Figure 3. (a) Phasor plot analysis in the soluble fraction of 25QHtt, 109QHtt, and 109QHtt in the presence of 8R10Q in live neuro2a cells. (b) Phasor plot analysis in the aggregated fraction of 109QHtt and 109QHtt in the presence of 8R10Q. Pixels highlighted in purple correspond to the points selected within the red circle in each phasor plot. Scale bar: 10 µm.

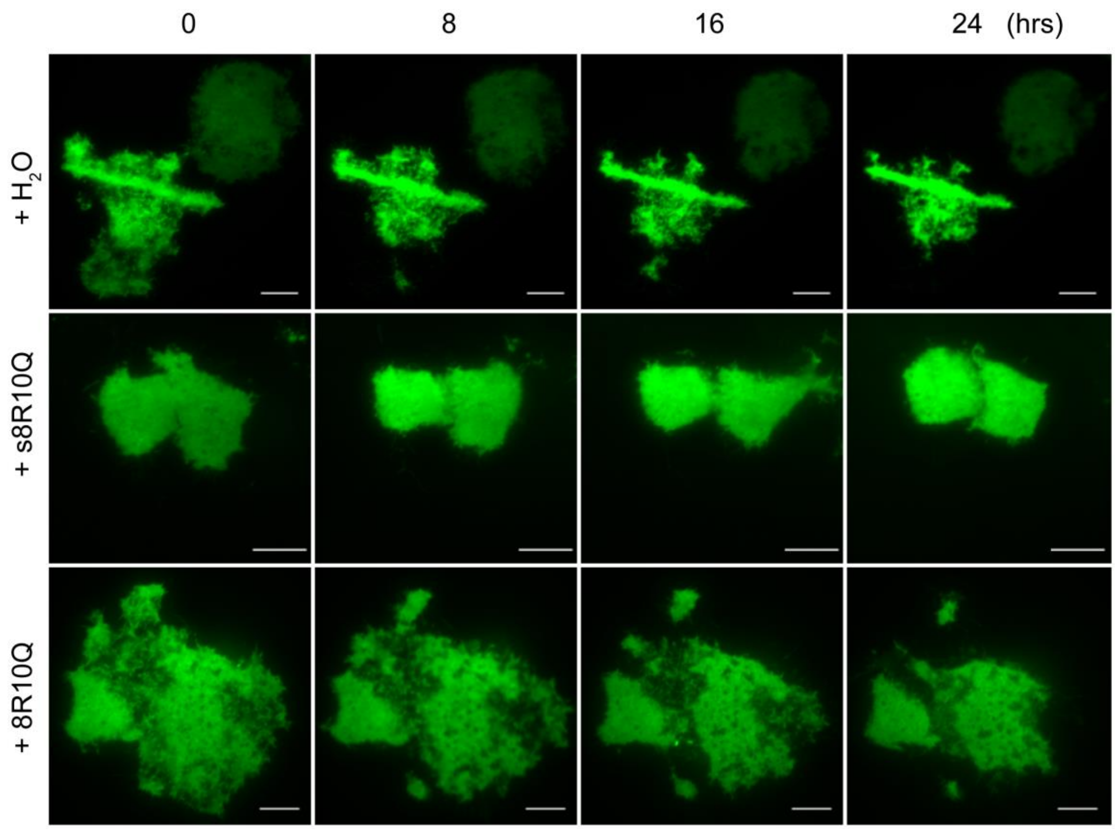

Supplementary Figure 4. TIRF micrographs of the Neuro2a expressing 25QHtt treated with water or peptides as indicated at various time points. Note no aggregation formation was observed with various peptide treatments. Scale bars, 10 µm.

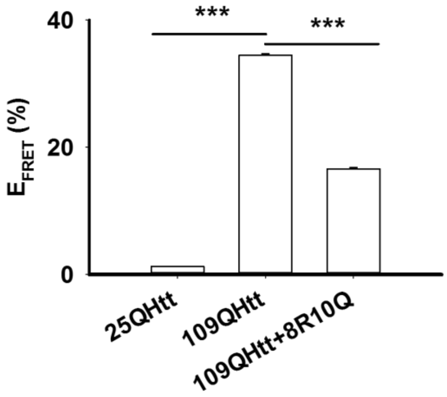

Supplementary Figure 5. Quantitative FRET efficiency (E_{FRET}) of the soluble fraction in 25QHtt, aggregated fraction of 109QHtt, and aggregated fraction of 109QHtt after 8R10Q treatment (details in Material and Methods). Data was shown as mean ± s.e.m, n=12. Statistics were done with one-way ANOVA followed by Duncan's Multiple Range test. ***p<0.001.

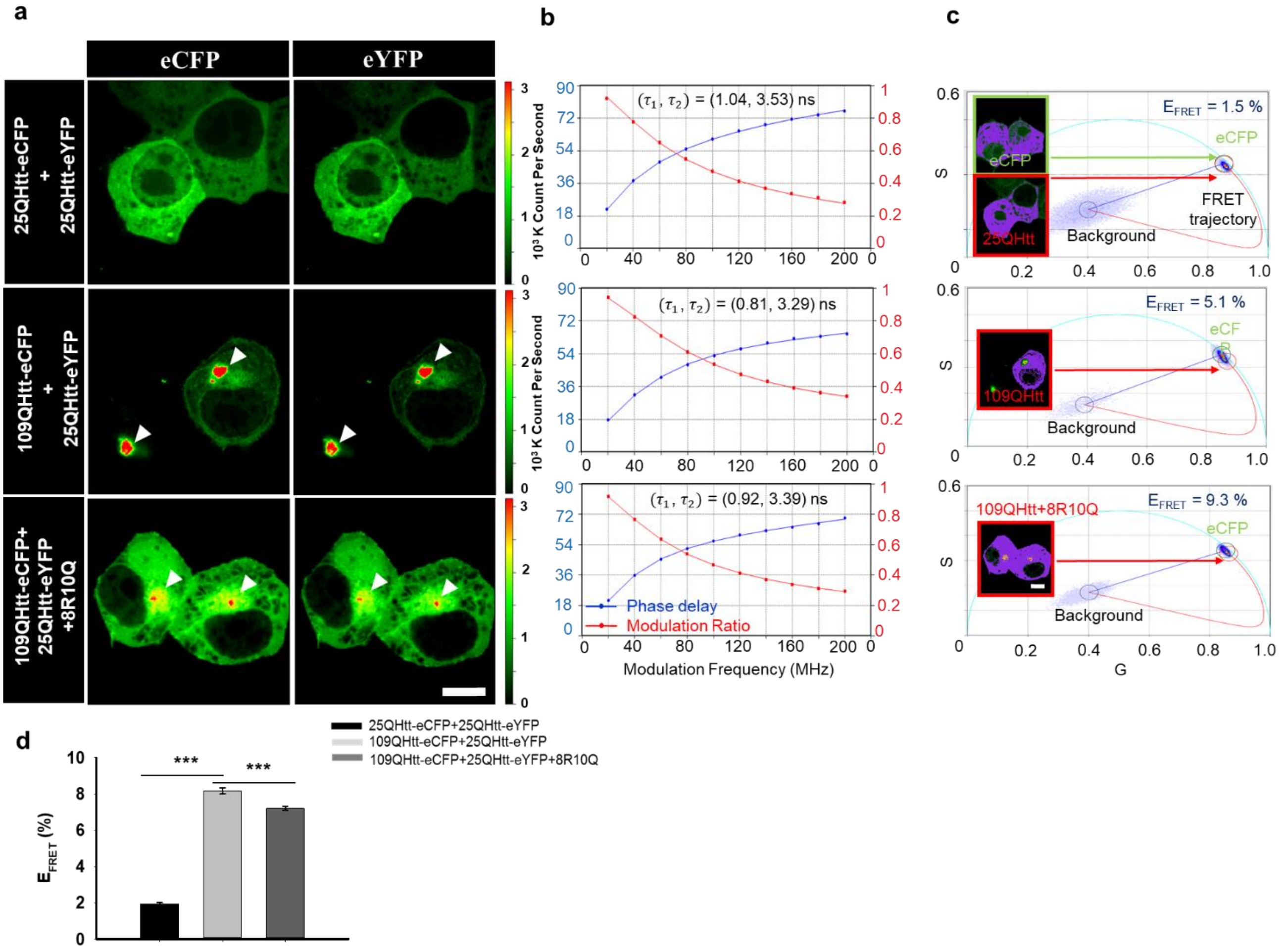

Supplementary Figure 6. 8R10Q decreased the compactness of heterogeneous mHTT oligomers and shifted the oligomerization process in Neuro2a cell. (a) Fluorescence intensity images of Neuro2a cells co-expressing 25QHtt-eCFP and 25Q-eYFP (top panel), 109QHtt-eCFP and 25QHtt-eYFP (middle panel), and 109QHtt-eCFP and 25QHtt-eYFP treated with 8R10Q (bottom panel), respectively. (b) Donor multi-frequency FLIM data of the soluble fraction in Supplementary Figure 11a was fitted with double exponential decay model. Additional information was summarized in Supplementary Table 5. (c) The phasor plot of the soluble fraction in Supplementary Figure 11a and the corresponding FRET efficiency (0-100%) from the curved trajectory. Pixels highlighted in purple correspond to the phasors within green or red circle. (d) Quantitative FRET efficiency (E_{FRET}). Data was shown as mean ± s.e.m, n=12. Statistics were done with one-way ANOVA followed by Duncan's Multiple Range test. ***p<0.001. Scale bar: 10 μm.

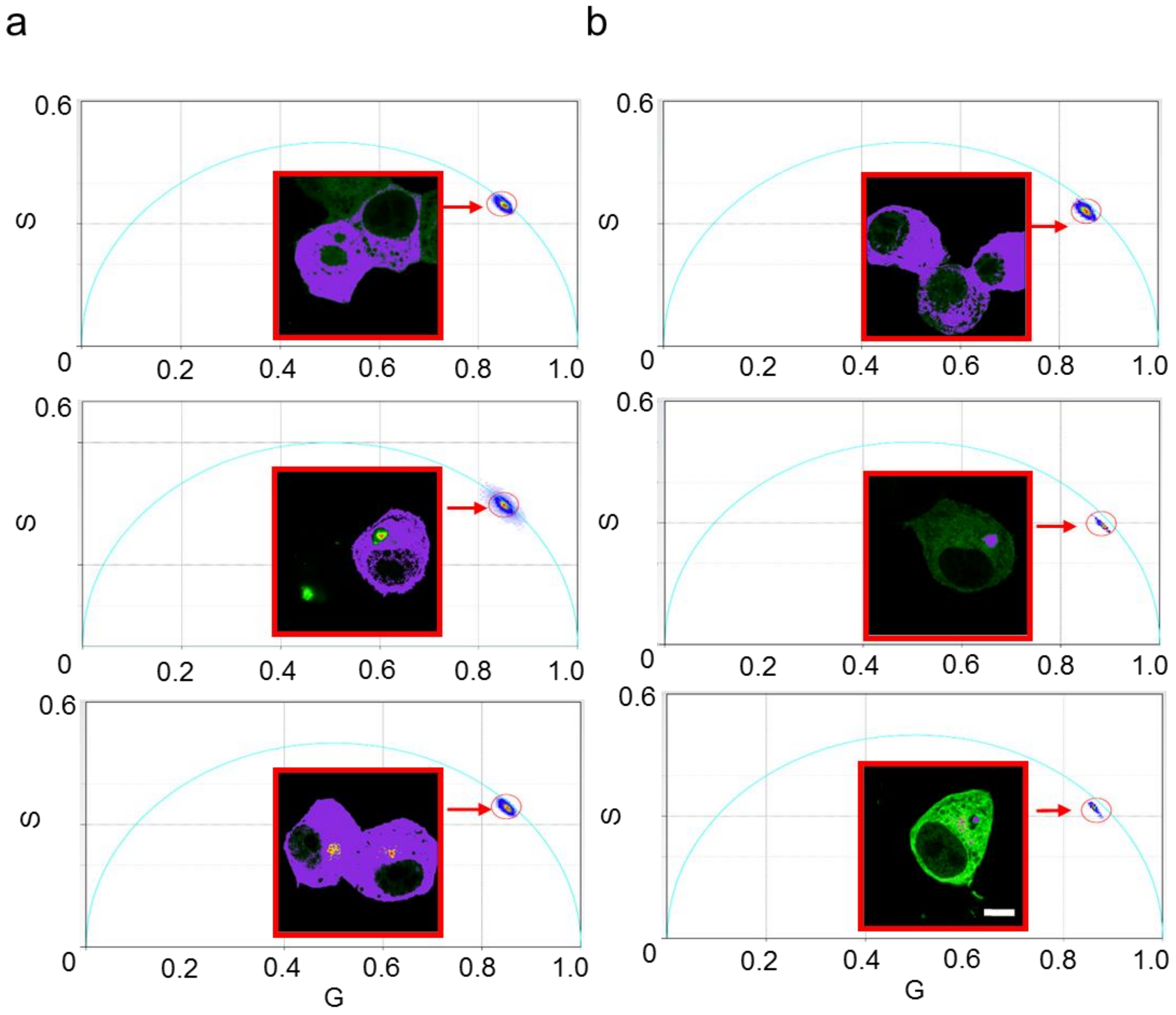

Supplementary Figure 7. Phasor plot analysis of the (a) soluble and (b) aggregated fraction of heterogeneous mHtt (109QHtt-eCFP and 25QHtt-eYFP) in the presence and absence of 8R10Q. Top panel: Neuro2a cells co-expressing 25QHtt-eCFP and 25Q-eYFP. Middle panel: Neuro2a cells co-expressing 109QHtt-eCFP and 25QHtt-eYFP without the treatment of 8R10Q. Bottom panel: Neuro2a cells co-expressing 109QHtt-eCFP and 25QHtt-eYFP treated with 8R10Q. Pixels highlighted in purple correspond to the points selected within the red circle in each phasor plot. Scale bar: 10 μm.

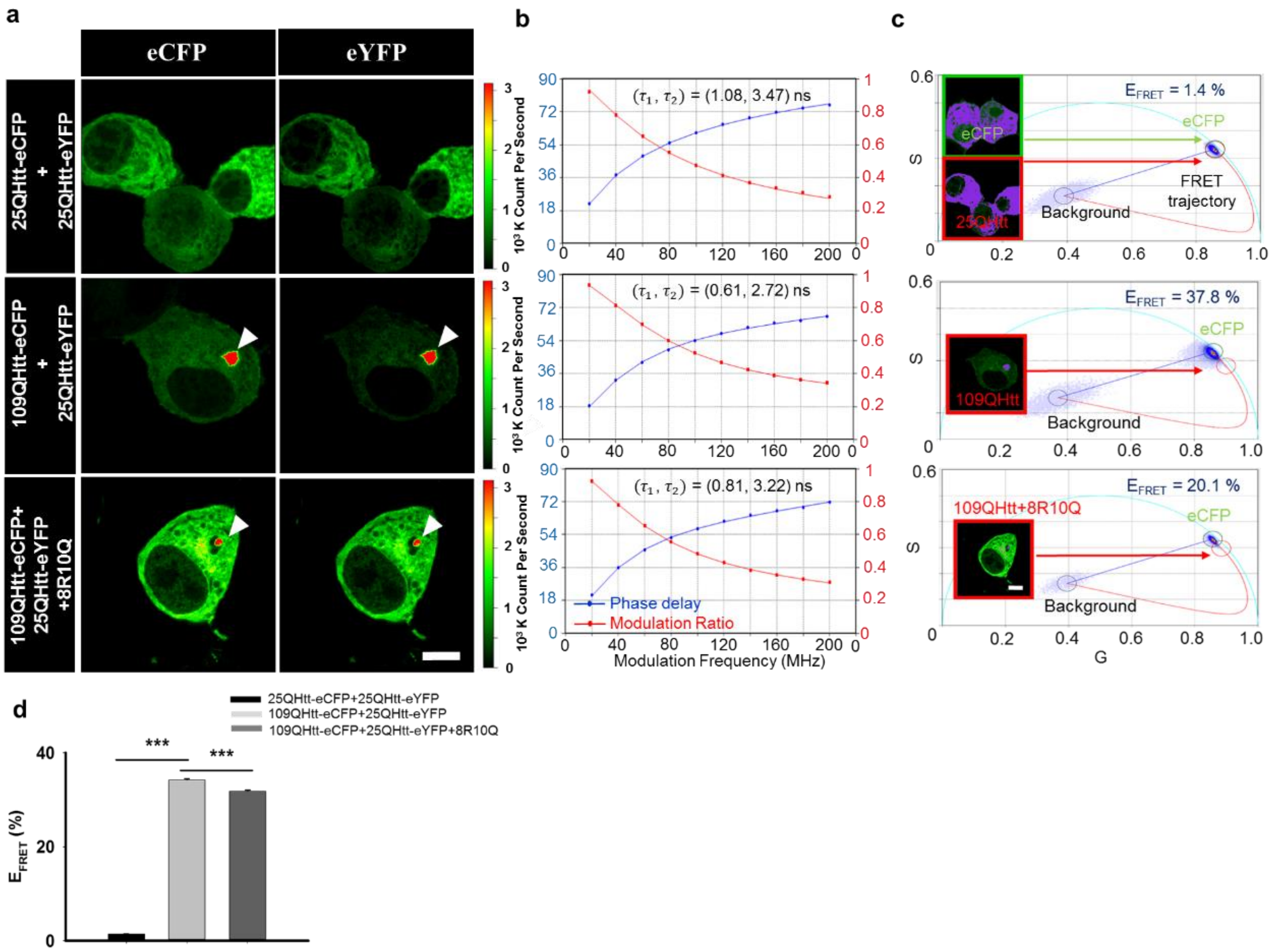

Supplementary Figure 8. Treatment of 8R10Q prevented large heterogeneous mHTT inclusion formation in Neuro2a cell. (a) Fluorescence intensity images of living neuro2a cells co-expressing 25QHtt-eCFP and 25Q-eYFP (top panel), 109QHtt-eCFP and 25QHtt-eYFP without the treatment of 8R10Q (middle panel) and 109QHtt-eCFP and 25QHtt-eYFP treated with 8R10Q (bottom panel), respectively. (b) Donor multi-frequency FLIM data of the aggregated fraction in Supplementary Figure 8a was fitted with double exponential decay model. Additional information was summarized in Supplementary Table 6. (c) The phasor plot of the aggregated fraction in Supplementary Figure 8a and the corresponding FRET efficiency (0-100%) from the curved trajectory. Pixels highlighted in purple correspond to the phasors within green or red circle. (d) Quantitative FRET efficiency (E_{FRET}). Data was shown as mean ± s.e.m, n=12. Statistics were done with one-way ANOVA followed by Duncan's Multiple Range test. ***p<0.001. Scale bar: 10 μm.

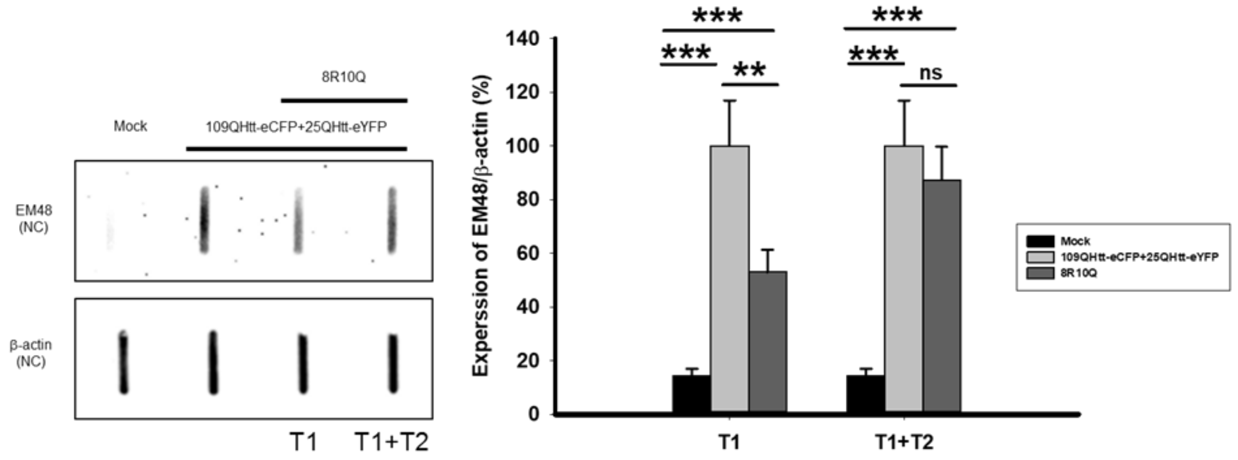

Supplementary Figure 9. Filter trap assay of heterogeneous mHtt (109QHtt-eCFP and 25QHtt-eYFP) in the presence and absence of 8R10Q in Neuro2a cell. Left panel: filter trap assay of heterogeneous mHtt treated with H_2O (Mock) or 8R10Q at the 8th hr (T1), the 24th hr (T2), or treated twice (T1+T2) after transfection. Samples were probed with either EM48 or β-actin antibody, and quantified by Image J (Right panel). All data was shown in mean ± s.e.m., N=3. Statistics were done with one-way ANOVA followed by Duncan's Multiple Range test. ***p<0.001; ns: not significant.

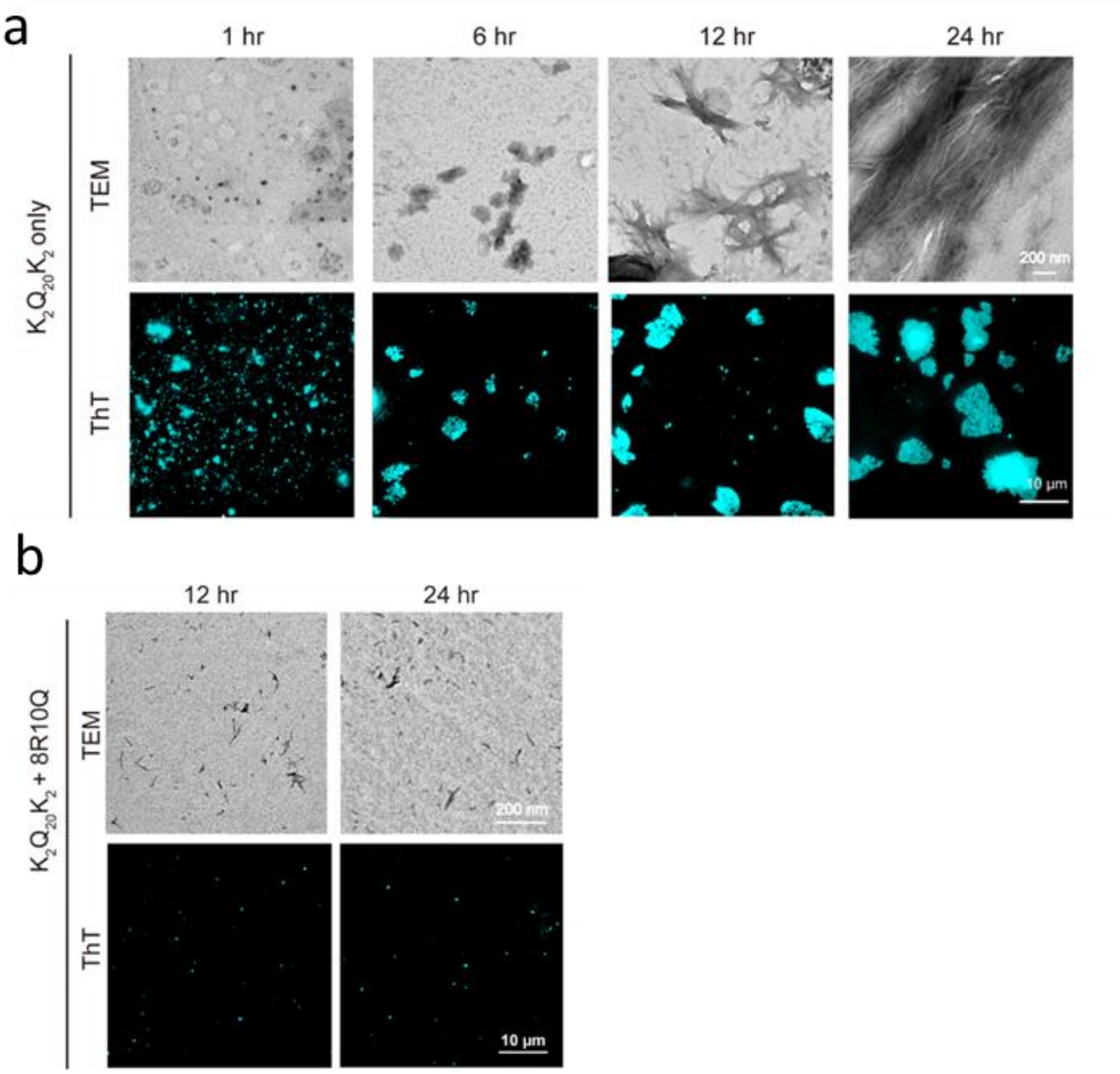

Supplementary Figure 10. Amphiphilic peptides halt the fibrillization process to form short fragmented aggregate species with low ThT intensity. (a) Upper lane: TEM imaging of $K_2Q_{20}K_2$ peptide incubated after 1, 6, 12, 24 hr. Scale bar: 200 nm. Lower lane: $K_2Q_{20}K_2$ were stained with ThT and imaged by TIRFM at the indicated time points. Scale bar: 10 μm. (b) Upper lane: TEM imaging of $K_2Q_{20}K_2$ peptide with 8R10Q incubated after 12 and 24 hr. Scale bar: 200 nm. Lower lane: $K_2Q_{20}K_2$ were stained with ThT and imaged by TIRFM at the indicated time points. Scale bar: 10 μm.

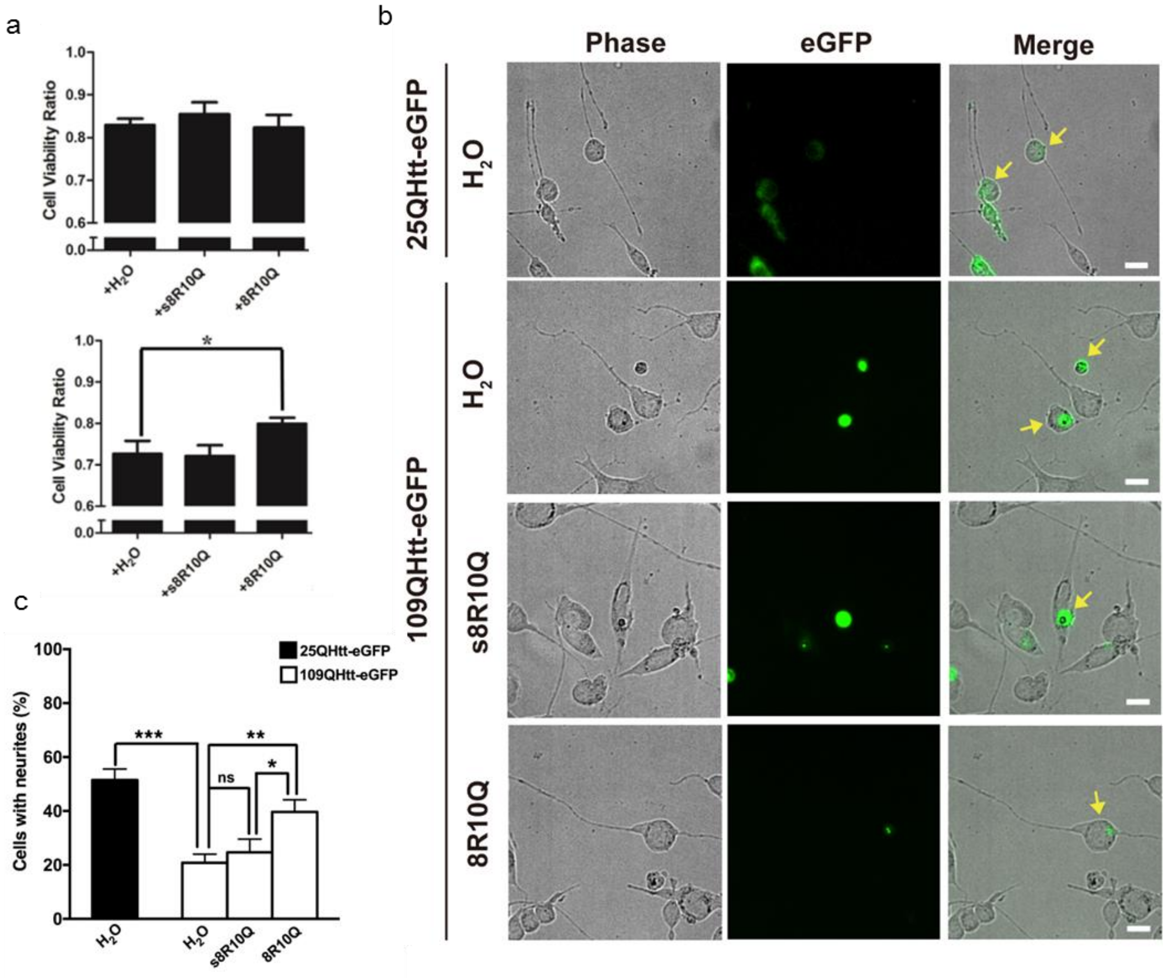

Supplementary Figure 11. 8R10Q peptide increased cell viability and neurite outgrowth in 109QHtt-eGFP expressing cells. (a) Cell viability assay by MTT in Neuro2a cells expressing 25QHtt- (top) or 109QHtt- (bottom) treated with the indicated peptides. (b) Micrographs of the phase contrast and fluorescent images of retinoic acid-differentiated Neuro2a cells expressing 25QHtt-eGFP or 109QHtt-eGFP treated with indicated peptide. Only cells expressing HTT-eGFP (yellow arrow) and process twice as long as its cell body were counted as neurite-bearing cells. (c) Quantification of the percentage of neurite-bearing cells. Percentage of cells with neurites was significantly decreased in 109QHtt-eGFP expressing cells compared with 25QHtt-eGFP controls. The experiments were conducted in triplicates and repeated twice. All data were shown as mean ± s.d. Statistics done with one-way ANOVA, *p<0.05; **p<0.01; ***p<0.001, ns: not significant.

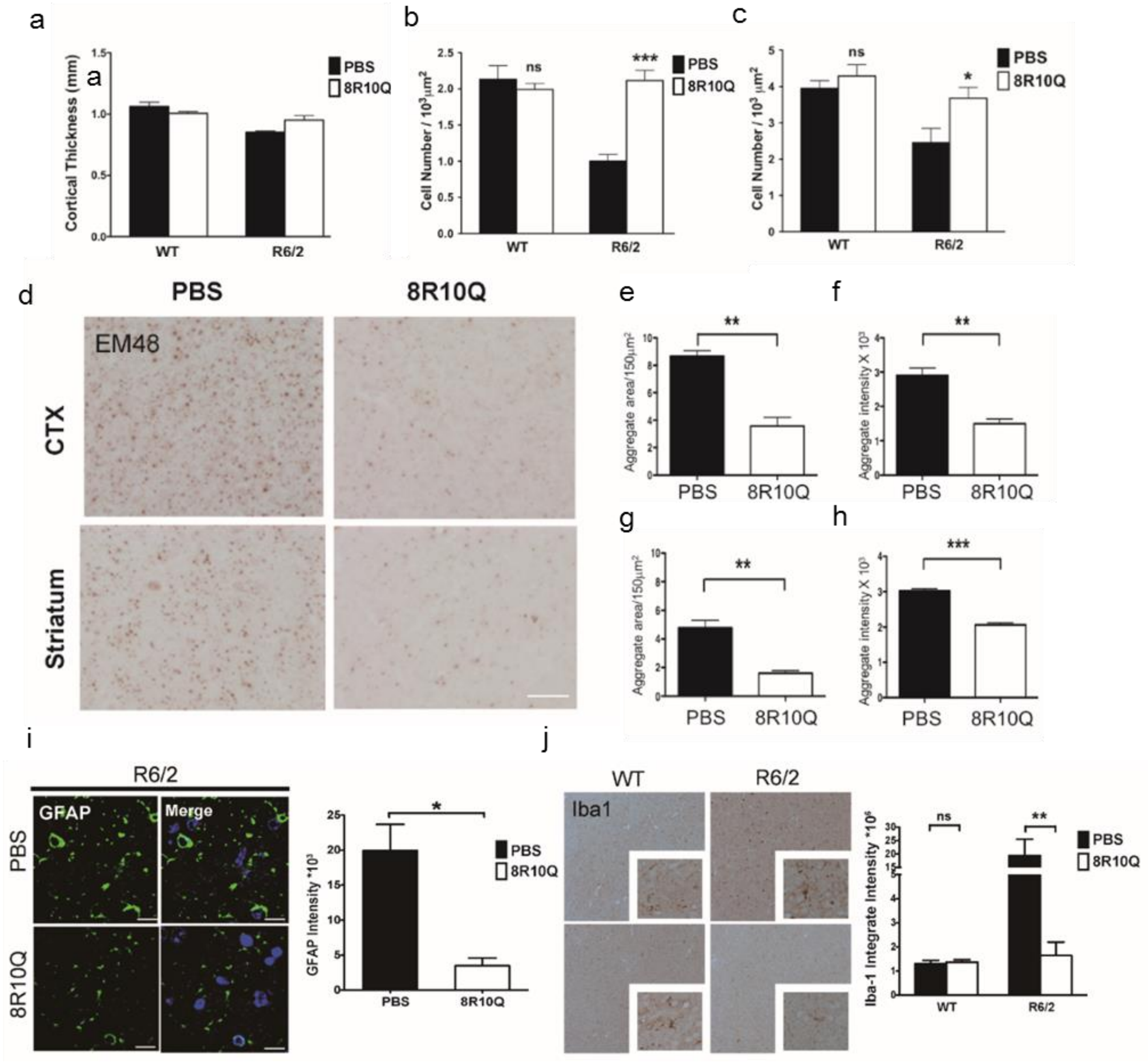

Supplementary Figure 12. Decrease in neuronal damage and glial pathology in 13-week-old R6/2 mice by 8R10Q peptide treatment. (a) Quantitation of the cortical (CTX) thickness of WT and R6/2 mice. Note the decrease in cortical thickness in R6/2 mice which was reversed by the 8R10Q peptide. (b) and (c) represent the quantitation of neuron numbers in Nissl stain sections of cortex (CTX) and striatum in WT and R6/2 mice, respectively. Note the decrease in neuron numbers in cortex and striatum was significantly reversed by 8R10Q. N=3/group, each bar was average of 5 sections. (d) Micrographs and the corresponding quantitation (e-h) of the immunostained sections of cortex (CTX) and striatum with EM48 antibody. The brown dots were the mHTT aggregates. The left subpanels (e-g) were the quantitative data on total area occupied by the aggregates, and the right (f-h) showed the intensity of individual aggregates. (i) Left: Micrographs of the immunofluorescent stained sections of the cortex of R6/2 mice with anti-GFAP antibody. Right: The quantitation of the GFAP intensity showed a significant decrease in 8R10Q treated mice. (j) Left: Micrographs of the

immunostained sections of cortex (CTX) and striatum of WT and R6/2 mice with anti-Iba1 antibody. Right: Quantitation of Iba1 intensity revealed an obvious increase in Iba1 immunoreactivity in R6/2 mice which was reversed by the 8R10Q. N = 3/group. Each bar represented an average of 15 sections/mouse group. All data were shown as mean ± s.d.; statistics all done with Student t test for, *$p<0.05$; **$p<0.01$; ***$p<0.001$, ns: not significant.

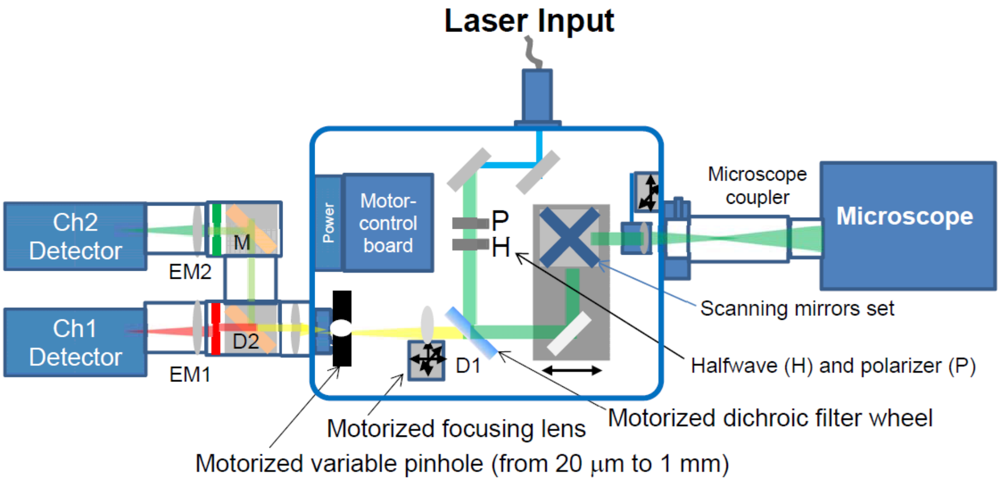

Optics	Description
**D1**	Dichroic beam splitter mirror to reflect laser beam and reflect sample emissions
**D2**	Dichroic beam splitter mirror to separate emission light between Ch1 and Ch2
**EM1**	Emission filter for Ch1
**EM2**	Emission filter for Ch2

Supplementary Figure 13. Schematic of ISS Q2 laser scanning confocal nasoscope for the frequency-domain fluorescence lifetime imaging.

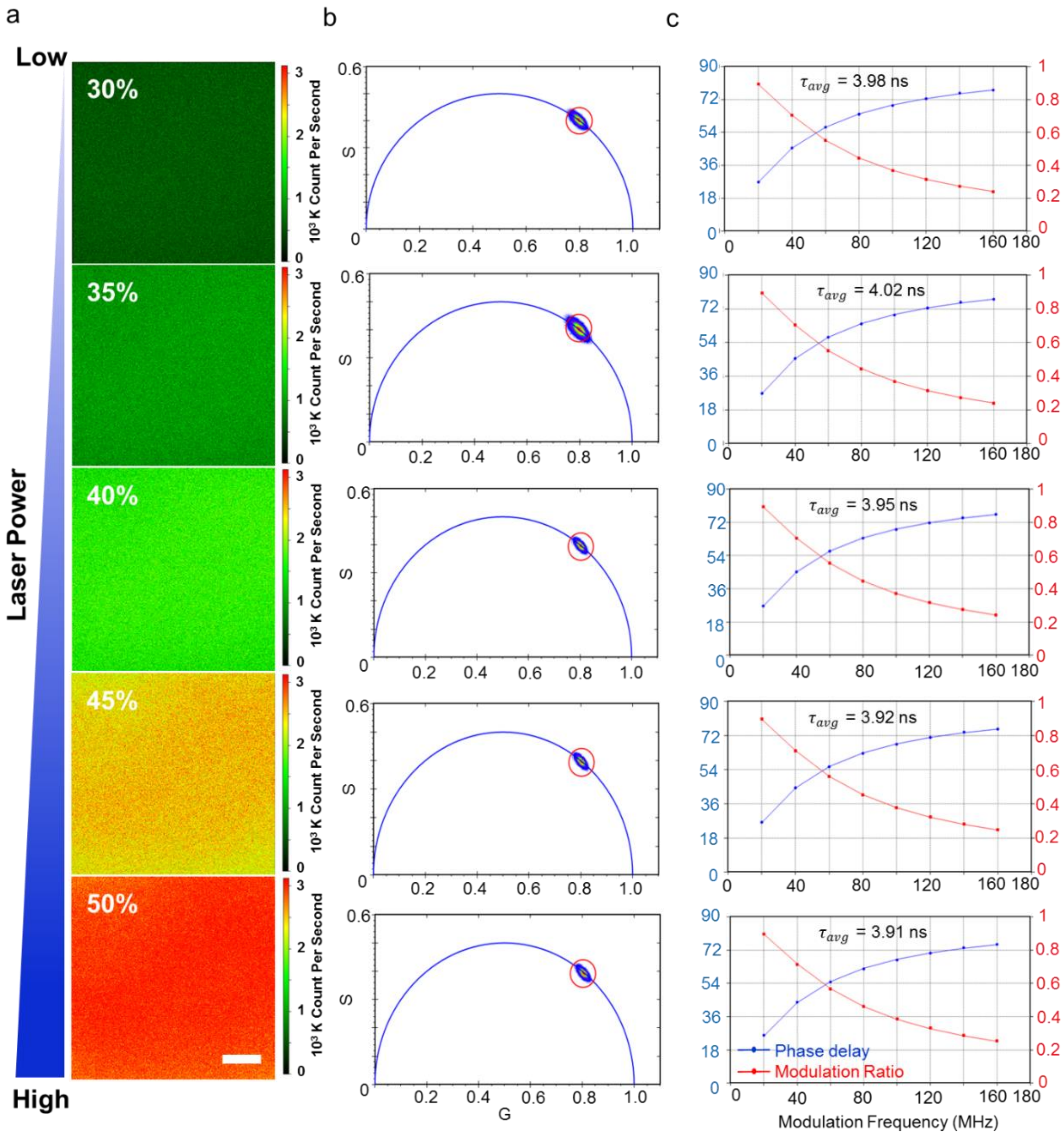

Supplementary Figure 14. Frequency-domain FLIM measurement of fluorescein with different laser powers. (a) Fluorescence intensity images (512 × 512 pixel) of 1 μM fluorescein in ddH_2O water were grabbed by frequency-domain FLIM at 20 MHz with 488 nm laser at different power (30%, 35%, 40%, 45% and 50% of laser power). (b) Phasor plot analysis of Supplementary Figure 4a. Pixels in each figure of Supplementary Figure 14a corresponded to the points selected within the red circle in each phasor plot. A threshold was applied to eliminate background noise, and a Gaussian 2D spatial convolution filter was used for smoothing. The phasor distribution for fluorescein molecules fell on the universal semicircle, representing a single exponential lifetime. (c) Multi-frequency FLIM data of Fluorescein was fitted with single exponential decay model. Scale bar: 10 μm.

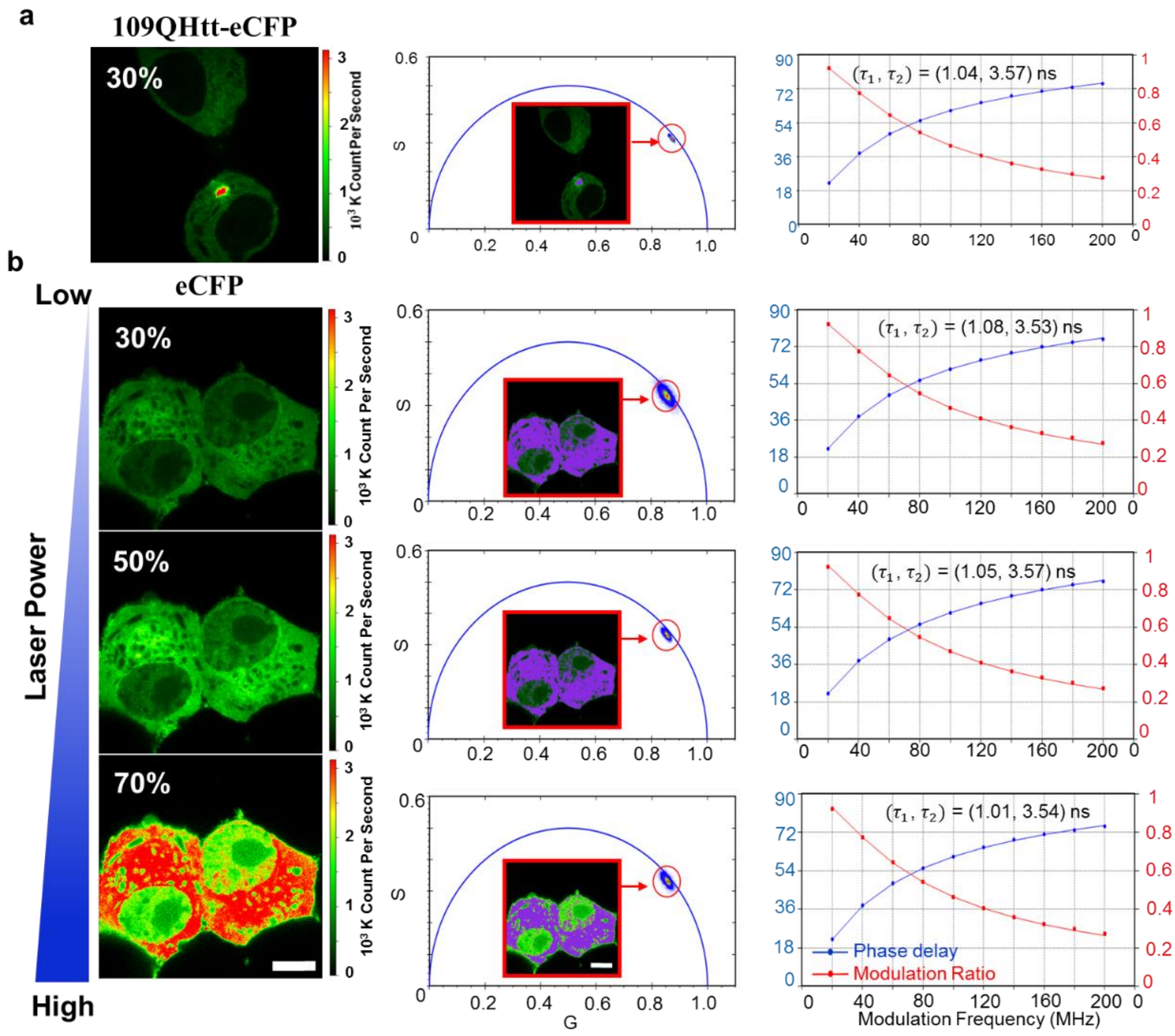

Supplementary Figure 15. Frequency-domain FLIM measurements of Neuro2a cells expressing 109QHtt-eCFP or eCFP alone construct with different laser power. Fluorescence intensity images (512 × 512 pixel, left column), phasor plot analysis (middle column), and multi-frequency lifetime fitting curves (right column) of living Neuro2a cells expressing (a)109QHtt-eCFP or (b) eCFP alone measured by frequency-domain FLIM with 440 nm laser of different power (30%, 50% and 70% of laser power) at 20 MHz. For the phasor plot analysis, pixels highlighted in purple correspond to the points selected within the red circle in each phasor plot. Since the phasor distribution for donor molecules fell within the universal semicircle, the lifetime should be fitted with multi-exponential lifetime. Donor multi-frequency FLIM data was better fitted by double exponential decay model. Additional information was summarized in Supplementary Table 7. Scale bar: 10 μm.

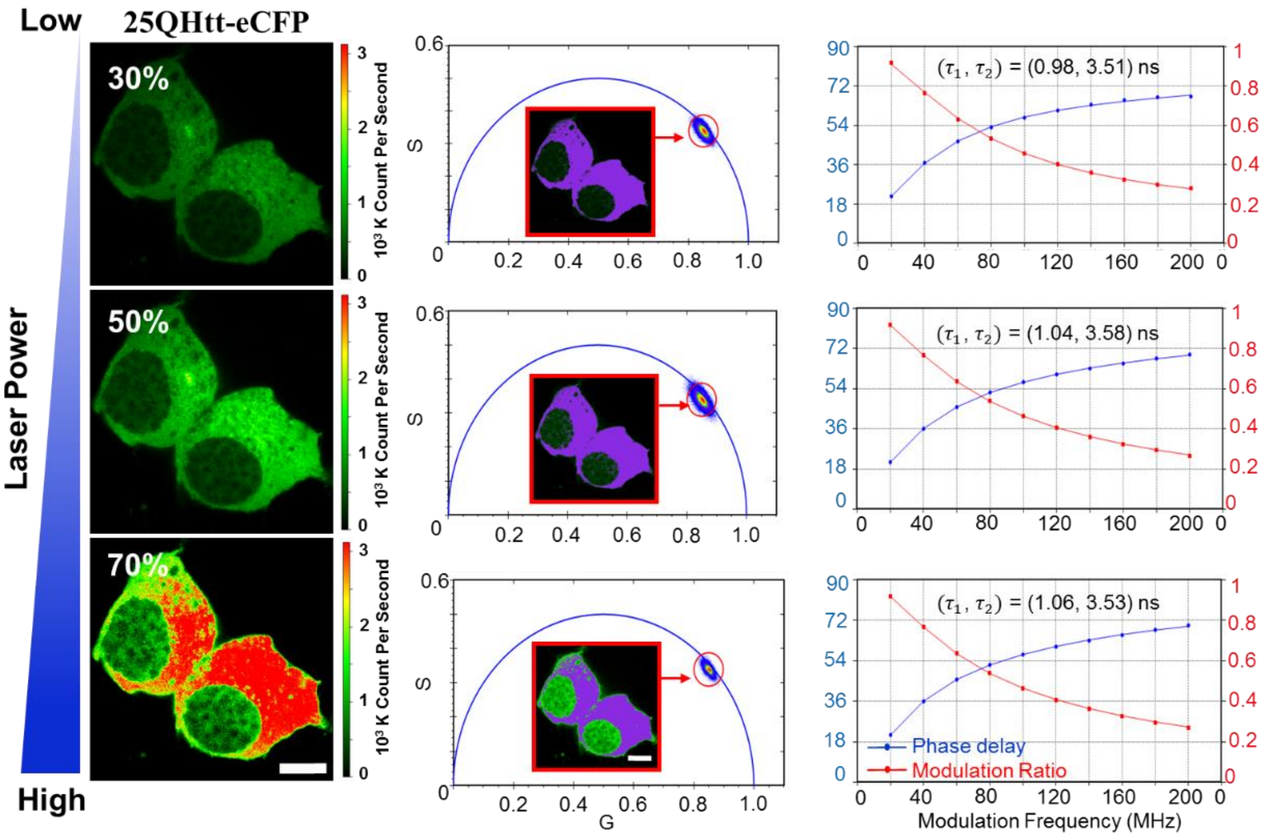

Supplementary Figure 16. Frequency-domain FLIM measurements of Neuro2a cells expressing 25QHtt-eCFP construct with different laser power. Fluorescence intensity images (512 × 512 pixel, left column), phasor plot analysis (middle column), and multi-frequency lifetime fitting curves (right column) of living Neuro2a cells expressing 25QHtt-eCFP measured by frequency-domain FLIM with 440 nm laser of different power (30%, 50% and 70% of laser power) at 20 MHz. For the phasor plot analysis, pixels highlighted in purple correspond to the points selected within the red circle in each phasor plot. Donor multi-frequency FLIM data was fitted by double exponential decay model. Additional information was summarized in Supplementary Table 8. Scale bar: 10 μm.

Supplementary Table 1. The sequence and biochemical properties of the amphiphilic peptides

Peptide	Sequence	Formula	MALDI-TOF		Solubility (DMEM/ 10%FBS)
			Calculated	Found	
**8R**	NH_2-$(R)_8$-W-D-$CONH_2$	$C_{63}H_{113}N_{35}O_{13}$	1567.8	1568.0	Soluble
**8R10Q**	NH_2-$(R)_8$-W-D-$(Q)_{10}$-$CONH_2$	$C_{113}H_{193}N_{55}O_{33}$	2849.2	2849.2	Soluble
**s8R10Q**	NH_2-RQQRRQQDQRQWQRQRQQRR-$CONH_2$	$C_{113}H_{193}N_{55}O_{33}$	2849.2	2849.5	Soluble

s8R10Q: scrambled 8R10Q

Supplementary Table 2. The fitted fluorescence lifetime of the soluble fraction in Fig. 1d.

	τ_1 (ns)	f_1	τ_2 (ns)	f_2	χ^2
**25QHtt**	1.07	0.199	3.51	0.801	0.31
**109QHtt**	0.85	0.184	3.31	0.816	0.42
**109QHtt+8R10Q**	0.94	0.202	3.45	0.798	0.35

The fluorescence lifetime of the donor (τ_1 and τ_2) was fitted by double exponential decay model. f_1 and f_2 represent the corresponding fraction in τ_1 and τ_2.

Supplementary Table 3. The fitted fluorescence lifetime of the soluble fraction in Supplementary Figure 2.

	τ_1 (ns)	f_1	τ_2 (ns)	f_2	χ^2
**25QHtt-eCFP**	1.02	0.195	3.55	0.805	0.46
**25QHtt-eCFP + 25QHtt-eYFP**	1.03	0.209	3.51	0.791	0.51

The fluorescence lifetime of the donor (τ_1 and τ_2) was fitted by double exponential decay model. f_1 and f_2 represent the corresponding fraction in τ_1 and τ_2.

Supplementary Table 4. The fitted fluorescence lifetime of the aggregated fraction in Fig. 2e.

	τ_1 (ns)	f_1	τ_2 (ns)	f_2	χ^2
**109QHtt**	0.58	0.245	2.51	0.755	0.33
**109QHtt+8R10Q**	0.79	0.192	3.14	0.808	0.41

The fluorescence lifetime of the donor (τ_1 and τ_2) was fitted by double exponential decay model. f_1 and f_2 represent the corresponding fraction in τ_1 and τ_2.

Supplementary Table 5. The fitted fluorescence lifetime of the aggregated (109QHtt-eCFP) and soluble (eCFP alone) fraction in Supplementary Figure. 15

	τ_1 (ns)	f_1	τ_2 (ns)	f_2	χ^2
**109QHtt-eCFP**	1.04	0.189	3.57	0.811	0.39
**eCFP 30%**	1.08	0.202	3.53	0.798	0.48
**eCFP 50%**	1.05	0.19	3.57	0.801	0.31
**eCFP 70%**	1.01	0.197	3.54	0.803	0.46

The fluorescence lifetime of the donor (τ_1 and τ_2) was fitted by double exponential decay model. f_1 and f_2 represent the corresponding fraction in τ_1 and τ_2.

Supplementary Table 6. The fitted fluorescence lifetime of the soluble fraction in Supplementary Figure. 16

	τ_1 (ns)	f_1	τ_2 (ns)	f_2	χ^2
**25QHtt-eCFP 30%**	0.98	0.192	3.51	0.808	0.34
**25QHtt-eCFP 50%**	1.04	0.204	3.58	0.796	0.37
**25QHtt-eCFP 70%**	1.06	0.201	3.53	0.799	0.46

The fluorescence lifetime of the donor (τ_1 and τ_2) was fitted by double exponential decay model. f_1 and f_2 represent the corresponding fraction in τ_1 and τ_2.

Supplementary Notes

Phasor Plot for TCSPC FLIM data. The phasor plot, also known as the AB plot or the polar plot (2-6), is a graphic representation of the raw FLIM data. Transforming FD or TCSPC FLIM data into the phasor plot is described below. In fluorescence lifetime imaging, the decay times of fluorophores are determined at each spatial location of an image. Assuming an infinite short excitation pulse (δ-function), an intrinsic fluoresce decay composed of N (N≥1) fluorescent species with distinct fluoresce lifetimes can be modeled by Eq. [1].

$$I(t) = I(0)\sum_{i=1}^{N}\alpha_i e^{-t/\tau_i}$$

[1]

Where $I(0)$ is the number of the instantly emitted photons at time zero; the coefficient α_i, called the pre-exponential factor, $I(0)\alpha_i$ is the amplitude and τ_i is the fluorescence decay time of the i-th component of the mixture. The amplitude weighted lifetime τ_α (often called apparent lifetime) given by Eq. [2]. The average lifetime τ_f is given by Eq. [3], where the fraction f_i is weighted in consideration of the ith lifetime ($\alpha_i\,\tau_i$).

$$\tau_\alpha = \sum_{i=1}^{N}\alpha_i\tau_i$$

[2]

$$\tau_f = \sum_{i=1}^{N} f_i\tau_i \qquad f_i = \frac{\alpha_i\tau_i}{\sum_{i=1}^{N}\alpha_i\tau_i} \qquad [3]$$

Due to the finite response of a system, the measured decay signal $I(t)$ is a convolution form of the intrinsic decay and the instrument response function (*IRF*) plus the noise $n(t)$, as shown in Eq. [4].

$$I'(t) = IRF\otimes I(t) + n(t) = IRF\otimes\left\{I(0)\sum_{i=1}^{N}\alpha_i e^{-t/\tau_i}\right\} + n(t) \qquad [4]$$

Thus, accurate FLIM data analysis typically requires the calibration of the IRF. In time-domain FLIM, the IRF can be measured by recording the scattered excitation light when using one-photon excitation, or the second-harmonic generation (SHG) signals for two-photon excitation by using a sample that yields strong SHG, such as urea crystal. In frequency-domain FLIM, the IRF is typically calibrated with a known fluorescence lifetime standard. In TCSPC FLIM, a decay trace *I'(t)* (see Eq. [4]) is recorded at each pixel location. To present the data in frequency domain, we take the Fourier Transform of Eq.[4] to get real and imaginary components as in Eq. [5].

$$\vec{F}(\omega) = \mathcal{F}(I'(t)) = \mathcal{F}(IRF\otimes I(t) + n(t)) = \mathcal{F}(IRF) * \mathcal{F}(I(t)) + \mathcal{F}(n(t)) \qquad [5]$$

Notice that in the Frequency domain, the IRF is a simple multiplication of the IRF vector and the noise is a simple addition of the noise vector. During the calibration procedure, we simply subtract the noise vector and divide the IRF vector to get the emission fluorescent vector. If the emission fluorescent vector is from the standard calibration single component dye, the phasor will be on the semi-circle after the calibration. The later measurement of the unknown sample will be always

noise and IRF free for finding the unknown fluorescent lifetime(s) as in Eq. [6].

$$g(\omega) = \frac{\int_0^\infty I(t)\cos(\omega t)}{\int_0^\infty I(t)} \qquad\qquad s(\omega) = \frac{\int_0^\infty I(t)\sin(\omega t)}{\int_0^\infty I(t)} \qquad [6]$$

Therefore, after calibration, each fluorescent decay trace (without IRF and noise) of each pixel can be plotted as a single point ("phasor") in the phasor plot by applying the sine and cosine transforms to the measured decay data, as shown by Eq. [6] and Figure 1. Where the modulation frequency ω is now called the laser repetition angular frequency and calculated by multiplying the laser repetition rate multiply with 2π. By taking Eq. [1] into Eq. [6] and solving the integrals, we can then derive the following relationships between the phasor and the lifetime, given by Eq. [7].

$$g(\omega) = \sum_{i=1}^{N} \frac{f_i}{1+\omega^2\tau_i{}^2} \qquad\qquad s(\omega) = \sum_{i=1}^{N} \frac{f_i\omega\tau_i}{1+\omega^2\tau_i{}^2} \qquad [7]$$

N is the numb[illegible]f the fluorescence lifetime τ_i.

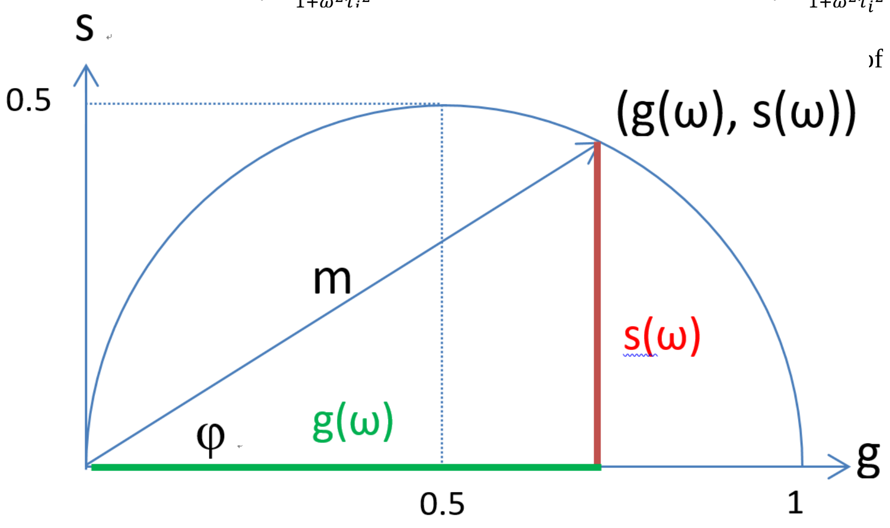

Figure 1. Mapping FD and TCSPC FLIM data to the phasor plot.

Phasor Plot for FD FLIM data. As in Figure 3, for one modulation frequency (ω), the FD FLIM data measurements at each pixel location are composed of both the phase delay (φ) and the amplitude modulation ratio (m). The FD FLIM data at each pixel can be mapped to a single point called "phasor" in the phasor plot through a transform defined by Eq. [8], where g(ω) and s(ω) represent the values at the two coordinates (g(ω), s(ω)) of the phasor plot.

$$g(\omega) = m\cos(\varphi) \qquad\qquad s(\omega) = m\sin(\varphi) \qquad [8]$$

In frequency-domain FLIM, this is done when calibrating the instrument prior to the data acquisition. There is no need to measure the IRF explicitly. The calibration procedure subtract the noise and divide the IRF and reveal the true fluorescent emission component(s). The universal semicircle for a single-lifetime species (N = 1), Eq. [8] is reduced to Eq. [9] and the lifetime can be directly determined by the coordinate values of a phasor. It is further derived that the two coordinates of a phasor representing a single-lifetime species must have the relationship given by Eq. [10]. The relationship is drawn as a semicircle curve centering at (G = 0.5, S = 0) with a radius of 0.5 in the phasor plot, and this semicircle curve is often called

the universal semicircle or the universal trajectory.

$$g(\omega) = \frac{1}{1+\omega^2\tau^2} \qquad s(\omega) = \frac{\omega\tau}{1+\omega^2\tau^2} \qquad \tau = \frac{1}{\omega}\left(\frac{s}{g}\right) \qquad [9]$$

$$(g(\omega) - 0.5)^2 + (s(\omega))^2 = (0.5)^2 \qquad [10]$$

Calibration and Fitting for Frequency Domain FLIM. In frequency domain, the calibration of the IRF is included in the standard sample calibration (10). Also, in Eq. [4], Eq.[5], and Eq.[6], we see the IRF and noise had been calibrated and the new measured phasor will present the unknown fluorescent only. For example, the standard sample can be fluorescein or Rhodamin 110, which has 4ns lifetime. The measured phase histogram of the standard lifetime sample includes the IRF and system noise. We take Fourier Transform of the IRF and system noise included phasor. That is, this phasor includes laser pulse time delay due to cable length, light path, detector response time, and also includes the IRF and the system noise. Then we calibrate this condition as 4ns on the semi-circle of the phasor plot. Then the system is then ready to measure any unknown sample, which will introduce a phase and modulation change only due to the sample fluorescent lifetime(s)

Fitting Equations, Components and Estimation of Frequency-Domain Phase and Modulation. After the Fourier Transform, we have fundamental frequency and harmonics. Each frequency has real and imaginary part after Fourier Transform [11]:

$$s(\omega) = \sum_{i=1}^{N} \frac{\alpha_i \omega \tau_i^2}{(1+\omega^2\tau_i^2)} / \sum_{i=1}^{N} \alpha_i \tau_i$$

$$g(\omega) = \sum_{i=1}^{N} \frac{\alpha_i \tau_i}{(1+\omega^2\tau_i^2)} / \sum_{i=1}^{N} \alpha_i \tau_i \qquad [11]$$

The measured phase and modulation has the relationship as below [12,13]:

$$\tan\varphi_\omega = s(\omega)/g(\omega)$$

$$m_\omega = \sqrt{(s^2(\omega) + g^2(\omega))} \qquad [12]$$

And fraction is defined as:

$$f_i = \alpha_i \tau_i / \sum_{i=1}^{N} \alpha_i \tau_i$$

[13]

And the Eq. [11] can be written as in Eq. [14].

$$s(\omega) = \sum_{i=1}^{N} f_i \frac{\omega \tau_i}{(1 + \omega^2\tau_i^2)}$$

$$g(\omega) = \sum_{i=1}^{N} f_i \frac{1}{(1+\omega^2 {\tau_i}^2)}$$

[14]

The fitting components will be τ_i and f_i. The Estimation is Levenberg-Marquardt algorithm for fitting the fraction (f_i) and lifetime (τ_i). The reduced chi-square χ^2 function at the pixel (h,k) is defined as:

$$\chi^2_{h,k} = \frac{1}{\nu}\left\{\sum_{j=1}^{N}\left[\frac{\varphi^j_{h,k}(\omega)-\varphi^C_{h,k}(\omega)}{\sigma_\varphi}\right]^2 + \sum_{j=1}^{N}\left[\frac{m^j_{h,k}(\omega)-m^C_{h,k}(\omega)}{\sigma_M}\right]^2\right\} \quad [15]$$

where:

N Total number of frequencies

ν Number of degrees of freedom. Since the number of data points is twice the number of frequencies, ν = 2N – p. p is the number of variables.

σ_ϕ , σ_M Uncertainties used in the phase and modulation values. It was found that the experimental result is not strongly dependent on σ_ϕ and σ_M .

$\phi_{i,j}(\omega)$ Measured frequency-dependent values of phase angle

$m_{h,k}(\omega)$ Measured frequency-dependent values of demodulation

$\phi^C_{h,k}(\omega)$ Calculated frequency-dependent values of phase angle at the pixel (h,k)

$m^C_{h,k}(\omega)$ Calculated frequency-dependent values of demodulation at the pixel (h,k)

Note: For consistency and ease of day-to-day interpretation, a constant error is used, σ_ϕ = 0.020 and σ_M = 0.004 for the calculation. The use of constant error does not introduce any ambiguity in data analysis since the accepting and rejecting a model is decided by the relative value of χ^2 . The χ^2 for one, two and three exponentials are compared. If χ^2 deceases by 50% or more as another component is added, it is better to include another component in the model. The χ^2 calculated in this way gives information about the degree of error in the experiment data. If the χ^2 is still quite high even for the best fitting option it would indicate that there is a systematic error or poor signal-to-noise ratio. Practically, rather than minimizing the χ^2 function at each pixel of the image, it is convenient to set an area including several pixels. VistaVision sofeware provides the values of the decay times and the relative fractional contribution in the defined area.

Reference

1. Ryan A. Colyer, Claudia Lee and Enrico Gratton 2007. *A novel fluorescence lifetime imaging system that optimizes photon efficiency.* Microsc Res Tech. Mar; 71(3):201-13.

2. Digman, M. A., V.R. Caiolfa, M. Zamai and E. Gratton 2008. *The phasor approach to fluorescence lifetime imaging analysis.* Biophys. J. 94, L14-6.

3. Stringari, C., A. Cinquin, O. Cinquin, M.A. Digman, P.J. Donovan and E. Gratton 2011. *Phasor approach to fluorescence lifetime microscopy distinguishes different metabolic states of germ cells in a live tissue.* Proc. Natl. Acad. Sci. U. S. A. 108, 13582-13587.

4. Redford, G. I. and R.M. Clegg 2005. *Polar plot representation for frequency-domain analysis of fluorescence lifetimes.* J. Fluoresc. 15, 805-815.

5. Clayton, A. H., Q.S. Hanley and P.J. Verveer 2004. *Graphical representation and multicomponent analysis of single-frequency fluorescence lifetime imaging microscopy data.* J. Microsc. 213, 1-5.

6. Hanley, Q. S. and A.H. Clayton 2005. *AB-plot assisted determination of fluorophore mixtures in a fluorescence lifetime microscope using spectra or quenchers.* J. Microsc. 218, 62-67.

7. Förster, T.1965. *Delocalized excitation and excitation transfer, In Modern quantum chemistry,* Sinanoglu, O., editors. Academic Press Inc. 93-137.

8. Clegg, R. M.1996. Fluoresce. *In Fluorescence resonance energy transfer. In Fluorescence imaging spectroscopy and microscopy,* Wang, X. F., Herman, B., editors. John Wiley & Sons Inc., New York. 179-251.

9. Sun, Y., R.N. Day and A. Periasamy 2011, *Investigating protein-protein interactions in living cells using fluorescence lifetime imaging microscopy.* Nat. Protoc. 6, 1324-1340

10. Suman Ranjit, Leonel Malacrida, David M. Jameson and Enrico Gratton 2018, *Fit-free analysis of fluorescence lifetime imaging data using the phasor approach.* Nat. Protoc. 13, Sep, 1979-2004
